# Intrinsically stretchable and transparent thin-film transistors based on printable silver nanowires, carbon nanotubes and an elastomeric dielectric

**DOI:** 10.1038/ncomms8647

**Published:** 2015-07-15

**Authors:** Jiajie Liang, Lu Li, Dustin Chen, Tibor Hajagos, Zhi Ren, Shu-Yu Chou, Wei Hu, Qibing Pei

**Affiliations:** 1 Department of Materials Sciences and Engineering, Henry Samuli School of Engineering and Applied Science, University of California, Los Angeles, California 90095, USA.

## Abstract

Thin-film field-effect transistor is a fundamental component behind various mordern electronics. The development of stretchable electronics poses fundamental challenges in developing new electronic materials for stretchable thin-film transistors that are mechanically compliant and solution processable. Here we report the fabrication of transparent thin-film transistors that behave like an elastomer film. The entire fabrication is carried out by solution-based techniques, and the resulting devices exhibit a mobility of ∼30 cm^2^ V^−1^ s^−1^, on/off ratio of 10^3^–10^4^, switching current >100 μA, transconductance >50 μS and relative low operating voltages. The devices can be stretched by up to 50% strain and subjected to 500 cycles of repeated stretching to 20% strain without significant loss in electrical property. The thin-film transistors are also used to drive organic light-emitting diodes. The approach and results represent an important progress toward the development of stretchable active-matrix displays.

## Abstract (web summary)

Stretchable displays need stretchable electronics to control them. Here, the authors create transparent and deformable thin-film transistors comprised of silver nanowire-based electrodes, a carbon nanotube network channel and a polyurethane-co-polyethylene oxide dielectric made using solution-based techniques.

## Graphical abstract

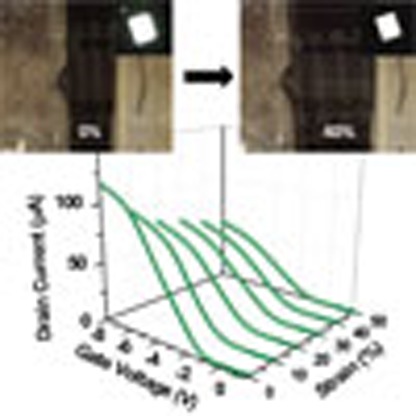

Wearable and biomedical electronic applications demand advanced materials and electronic devices to simultaneously possess deformability, solid state, light weight, visual transparency and low processing cost^1^,^2^,^3^,^4^,^5^. Stretchable thin-film field-effect transistor (TFT) would become a fundamental building component enabling a variety of stretchable electronic devices including displays, sensor arrays, thin-film circuits and identification tags for control, data-processing and communication. We have recently reported intrinsically stretchable solid-state organic light-emitting devices (OLED)^6^,^7^,^8^ and simple pixelated displays. For high-content information display, an intrinsically stretchable TFT array is also highly demanded to drive a display panel comprising a large number of pixels. Being transparent for the OLEDs and the TFTs would significantly expand the range of applications of the active-matrix displays and increase the aperture ratio of the display pixels. Developing a stretchable transparent TFT is thus a fundamental step toward this challenging yet exciting goal.

TFT is a three-terminal device comprised of source, drain and gate electrodes, a dielectric layer, a semiconductor layer and a substrate. Developing an intrinsically stretchable TFT entails development of all these materials with elastomeric stretchability. The requirements of visual transparency and printable process significantly increase the challenges in materials and process development. Traditionally, amorphous silicon and polysilicon are widely used as semiconductor channel for TFTs; evaporated metal films or indium tin oxide (ITO) and are preferred choices for the electrode materials; silicon oxide or high dielectric polymers are usually used for the dielectric materials. None of these materials is intrinsically stretchable, and most do not meet the requirements of being transparent and printable^9^. Advanced materials and processing techniques developed in recent years for flexible nanoelectronics employing metallic nanowires, semiconducting nanowires, single-walled carbon nanotubes (SWCNTs) and functional polymer-based dielectrics offer unique functionalities, promising performance and low-cost processing strategies that could be explored for the development of stretchable TFTs^10^,^11^,^12^,^13^.

To impart mechanical compliancy and robustness to these materials and the resulting TFTs, two general strategies have been employed to create TFTs capable of stretching without undergoing physical damage^13^. The first approach configures conventional brittle or rigid inorganics into specialized structural layouts and mechanical designs, such as wrinkled or buckled structures, to accommodate strains applied in the materials^14^,^15^,^16^,^17^,^18^,^19^,^20^,^21^. Non-stretchable device islands connected with stretchable conductors or wavy metal interconnects have realized high-performance integrated systems that consist of hundreds and even thousands of TFTs^14^,^15^. Kim *et al*.^17^ introduced non-coplanar mesh designs and serpentine interconnects for the array of stretchable complementary metal-oxide-semiconductor integrated circuits, enabling them to accommodate even demanding configurations such as corkscrew twists with tight pitch and linear stretching to strains ∼140%. Kaltenbrunner *et al*.^18^ fabricated organic field-effect transistors directly on ultrathin (1 μm) polymer foils which accommodate stretching up to 230% on pre-strained elastomers. A stretchable TFT combining stretchable graphene/SWCNT electrodes and a SWCNT network channel with a geometrically wrinkled Al_2_O_3_ dielectric layer has also been reported through this approach^19^. These reported devices can achieve high electrical performance and impressive stretchability, but lack visual transparency, and the fabrication process is cumbersome and incompatible with fully solution-printed process. The second strategy employs intrinsically stretchable materials and components to obtain fully (or intrinsically) stretchable TFT^22^,^23^,^24^,^25^. Several intrinsically stretchable TFTs have been reported, including a graphene-based TFT employing graphene as both the semiconducting channel and the source/drain electrodes, and an ion gel as the gate^24^, and an organic-based TFT using poly(3-hexylthiophene) as the channel, carbon nanotubes as the source/drain, polyurethane (PU) as dielectric and liquid metal as gate^22^. However, these devices are not solid state and suffer from either low electrical performance, opacity or limited stretchability. Despite great efforts being exerted in this field, so far no one has succeeded in demonstrating a fully stretchable, solid state, transparent TFT suitable for stretchable thin-film displays.

In the following, we report the successful development of such a device, using all solution-based printable processes. The work includes the following essential elements: (1) the stretchable TFT employs a silver nanowire (AgNW)-PU acrylate (PUA) composite as the stretchable transparent electrodes with high surface conductivity and low surface roughness, SWCNT network channel and a PU-*co*-polyethylene glycol (PU-*co*-PEG) elastomeric dielectric; the TFT has high mobility, modest ON/OFF current ratio (*I*_ON/OFF_), high peak ON current (*I*_ON_), large transconductance and relatively low operating voltages in unstretched state. (2) The TFT can be stretched by up to 50% while retaining a high mobility; at 20% strain, it can be stretched by 500 cycles; (3) the entire fabrication process is printable: the electrodes, semiconductor layer, dielectric layer and substrate are all processed from solutions at ambient conditions. (4) The TFT exhibits optical transmittance of >90% in the 450–1,100 nm wavelength range. (5) The TFT can drive an OLED in the full brightness range.

## Results

### Stretchable TFT fabricated by solution-based techniques

**Figure 1:**
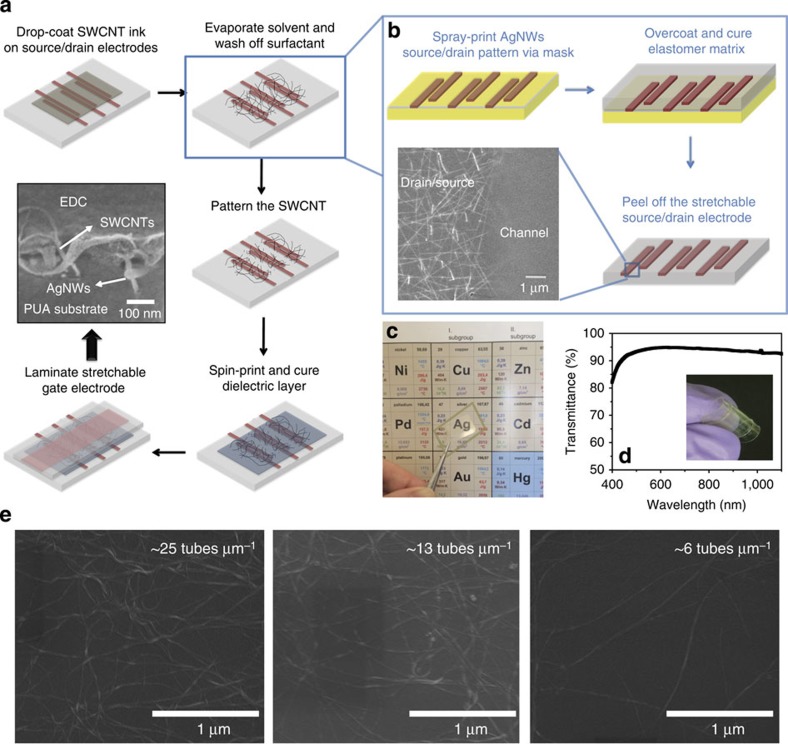
Fabrication process and the SEM and transmittance characterizations of the stretchable TFT. (**a**) Schematic illustration of the fabrication steps for a stretchable TFT. The optical microscopic image shows the AgNW-PUA composite source/drain electrodes covered with a SWCNT layer (100 μm channel length). (**b**) Fabrication process of AgNW-PUA composite source/drain electrodes. Inset: cross-sectional SEM image showing the SWCNTs embedded into and wrapped by the dielectric layer. (**c**) Optical image of a TFT array marked with a brown dash frame around the element ‘Ag'. (**d**) Optical transmittance of a TFT array. Inset photograph shows a folded TFT array. (**e**) SEM images of SWCNT network printed on AgNW-PUA composite source/drain electrodes, showing that the density can be controlled by the amount of SWCNT ink cast on the substrate.

The structure and fabrication process of the stretchable transparent TFT are illustrated in Fig. 1a. The first key step of the process is to form the patterned stretchable source/drain electrode, as illustrated in Fig. 1b. It started with spray-printing a layer of AgNWs from dispersion in methanol through a shadow mask on glass substrate. The opening of the mask defines the source/drain electrode pattern, with a channel length (*L*) of 100 μm and channel width (*W*) of 5,000 μm. A stretchable polymer PUA is chosen to transfer and embed the AgNW source/drain electrode into the surface layer of a rubbery matrix, according to a previously reported approach^8^. The inset scanning electron microscopy (SEM) image in Fig. 1b shows that the edge of the AgNW pattern is fairly smooth, which is essential to the reproducibility and stability of the TFT devices. The sheet resistance of AgNW-PUA composite electrode can be controlled in a wide range with varied transmittance. The electrode with 15 Ω sq^−1^ was chosen in this work for a compromised high conductivity, stretchability and optical transmittance^8^.

The second key step is to form a uniform channel material on the AgNW-PUA composite source/drain substrate which is hydrophobic. In this study, the active channel material was printed from drop-casting a certain amount of in-house formulated semiconductive SWCNT ink followed by drying and then rinsing with deionized water and isopropyl alcohol (IPA) alternatively. This semiconductive SWCNT ink was prepared by mixing Fluorosurfactant and propylene glycol with the 99+% semiconductive SWCNT solution (IsoNanotubes-S from NanoIntegris, Inc.) at an optimized ratio (detailed in Experimental part). The addition of Fluorosurfactant and propylene glycol enhances wetting and levelling control and of the SWCNT solution, and thus brings up uniform deposition of the SWCNT network on channel (Supplementary Fig. 1). The density of the printed SWCNT depends on the amount of SWCNT ink cast on the substrate. Three densities with estimated values of ∼25, 13 and 6 tubes μm^−1^ from three different casting amounts as shown in the SEM images from Fig. 1e were made and investigated in this study.

The third key aspect is to identify a stretchable and transparent dielectric layer with high dielectric constant and good mechanical compliance. For this purpose, PU-PEG copolymer with PU-*co*-PEG main chain and PEG side chain formed from siliconized urethane acrylate (CN990 from Sartomer Inc.), methoxy PEG monomethacrylate (SR550) and PEG diacrylate (SR259). The siliconized urethane acrylate oligomer was selected for its high stretchability and compatibility with the source/drain and gate composite electrode. The PEG diacrylate was selected for its high flexibility, low monomer viscosity which is important for printing, high dielectric constant^26^,^27^ and semicrystalline structure of its homopolymer^26^. Methoxy PEG monomethacrylate was selected to introduce side chains with its high polarity and chain mobility at room temperature. The precursor solution was overcast and spin-printed on the SWCNT/AgNW-PUA source/drain followed by UV-curing to form a thin elastomeric dielectric overcoating and wrapping around the SWCNTs. The cross-sectional SEM image of a typical device (inset in Fig. 1a) confirmed the dielectric-wrapped SWCNT structure. Various weight ratios of CN990:SR550:SR259 were studied, and a ratio of 1:1:1 was found to give the optimal overall performance of the resulting elastomeric dielectric copolymer (EDC) in terms of dielectric constant (as high as ∼13 at 12 Hz, see Supplementary Fig. 2), optical transmittance (>90% at 550 nm, see Supplementary Fig. 3a), and sufficient elongation at break (>50%, see Supplementary Fig. 3b). Moreover, as displayed in Supplementary Fig. 3c,d and Supplementary Table 1, the small loss factor (∼0.011), almost overlap of the stress-strain curves during loading and unloading, and high resilience value suggest small hysteresis, fast recovery and good elasticity for the EDC (see Supplementary Note 1).

Finally, the resulting EDC/SWCNT/AgNW-PUA source/drain structure was laminated with another 15 Ω sq^−1^ AgNW-PUA composite electrode as gate electrode on top of the dielectric layer to complete the TFT fabrication. Thanks to the amalgamation of all transparent components, the SWCNT-AgNW TFT arrays show high transparency, as displayed in Fig. 1c, where the element ‘Ag' of the periodic table can be clearly seen through a TFT array consisting of six TFT devices (marked with brown dash lines) in the optical image. Figure 1d depicts that the optical transmittance of the array is >90% in the 450–1,100 nm wavelength range.

### Electrical properties of the stretchable TFT

**Figure 2:**
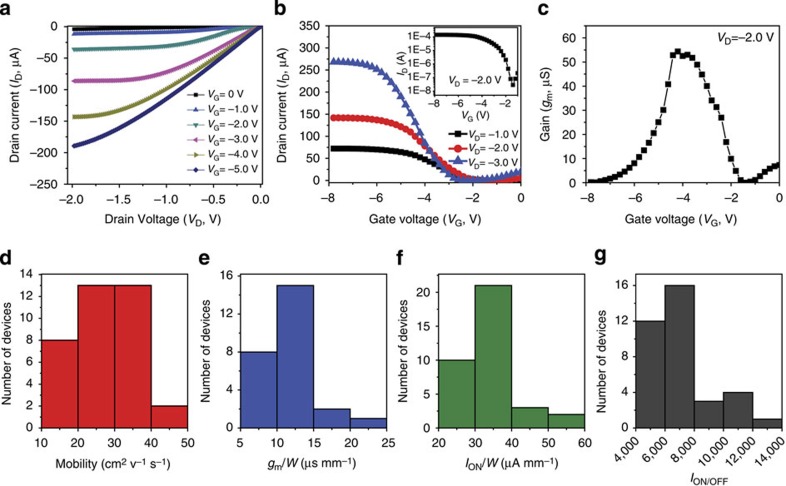
Electrical properties of a representative stretchable TFT and statistical variation studies in the electrical properties of 36 stretchable TFTs. (**a**) Output (*I*_D_–*V*_D_) characteristics of a typical SWCNT-AgNW TFT (*L*=100 μm and *W*=5,000 μm) with *V*_G_ from 0 to −5 V in 1 V steps. (**b**) Transfer (*I*_D_–*V*_G_) characteristics of the same device with *V*_D_ from −1.0 to −3.0 in 1.0 step. Inset, *I*_D_–*V*_G_ curve at *V*_D_=−2.0 V on a logarithmic scale. (**c**) Transconductance at *V*_D_=−2.0 V as a function of *V*_G_. (**d**–**g**) Histograms of TFTs showing the statistical distribution of (**d**) mobility, (**e**) unit width normalized transconductance, (**f**) unit width normalized *I*_ON_ and (**g**) the *I*_ON/OFF_.

Electrical characteristics of a typical SWCNT-AgNW TFT with a density of SWCNT about 25 tubes μm^−1^ are shown in Fig. 2a,b. All devices were tested in air at room temperature. The SWCNT (with 99+% semiconductive SWCNTs) used in this work has a length range from 300 nm to 5 μm. Channel length of >20 μm is reported to be required to decrease the probability of percolative transport through the metallic nanotubes in order to achieve *I*_ON/OFF_>10^4^ (refs 28, 29). A channel length of 100 μm and channel width of 5,000 μm are thus selected, but not optimized, in the present work for all device testing. A family of output curves appears to be linear at low fields with gate voltage stepped from −1 to −5 V. The specific contact resistance between the semiconducting SWCNT network and AgNW electrodes is calculated to be 0.219±0.043 Ω cm^2^ through the transmission line model measurements as detailed in Supplementary Fig. 4 and Supplementary Note 2 (refs 30, 31). The contact resistance between SWCNT and AgNW is higher than that between SWCNT and evaporated Ag electrode (0.02 Ω cm^2^)^30^,^31^. The work function of the AgNW is measured to be ∼4.90 eV by Kelvin probe force microscopy, which is higher than the typical work function of Ag (4.73 eV), indicating surface of AgNW partially covered by Ag_2_O. This surface oxide might have a dominant effect on the interface characteristics with SWCNTs, and thus increase the contact resistance. However, this contact resistance between AgNW and SWCNTs is still negligibly small compared with the channel resistances because of the large channel length used in this work^32^. Clear current saturation at high drain voltages due to pinch-off is also evident. Moreover, notable increase of the conductance of the channel with the increasing gate voltage from 0 to −5 V in 1 V steps manifests the effective gate modulation of the EDC.

From the transfer characteristics shown in Fig. 2b, one typical device exhibits a peak *I*_ON_ value of 140 μA and peak transconductance (*g*_m_) of 54.4 μS, corresponding to 28.0 μA mm^−1^ (*I*_ON_/*W*) and 10.9 μS mm^−1^ (*g*_m_/*W*) as normalized by the channel width at *V*_D_=−2.0 V. Well-defined ON-state and OFF-state currents were observed with a *I*_ON/OFF_ of 7,000 at *V*_D_=−2.0 V. A gate-sweep-rate-dependent hysteresis was also observed for the SWCNT-AgNW TFT devices (see Supplementary Fig. 5), which is typical for SWCNT-based devices (see Supplementary Note 3)^33^,^34^. The mobility of the device is extracted using the following equation: *μ*=(d*I*_D_/d*V*_G_)(*L*/*V*_D_*C*_i_*W*), where *μ* is the device mobility, *I*_D_ the drain current, *V*_G_ gate voltage, *L* channel length, *W* channel width and *C*_i_ specific capacitance of the elastomeric dielectric. *C*_i_ was measured from parallel-plate test structures with an average value of ∼16.7 nF cm^−2^ at 12 Hz with thickness being ∼1.1 μm. An unstretched device mobility of 32.5 cm^2^ V^−1^ s^−1^ is thus calculated from the transfer characteristics in Fig. 2b. However, SWCNTs are sparsely distributed in the channel (Fig. 1e), and the parallel-plate capacitor model overestimates the gate capacitance and underestimates the mobility. Using the cylindrical model that takes into consideration of the effect of electrostatic coupling between nanotubes to estimate the gate capacitance^19^,^35^,^36^, a higher mobility of 51.6 cm^2^ V^−1^ s^−1^ is obtained (see Supplementary Notes 4 and 5).

In 7 different batches for a total of 36 TFT devices fabricated, the yield was 85.7%. The *I*_ON/OFF_, peak *I*_ON_ value, *g*_m_ and mobility were measured in the ranges of 4,500–12,300, 8.6–23.0 μA, 32.3–72.1 μS and 19.3–43.1 cm^2^ V^−1^ s^−1^, respectively. Fig. 2d–f present the histograms of the statistical variations for these 36 devices in *I*_ON/OFF_, *I*_ON_/*W*, *g*_m_/*W* and mobility, respectively. The *I*_ON_/*W* and *g*_m_/*W* were measured at *V*_D_=−2.0 V, and the mobility was also extracted as described above at *V*_D_=−2.0 V. The average of *I*_ON/OFF_ ratio, *I*_ON_/*W* and *g*_m_/*W* and mobility is 7,200±1,200, 30.0±8.93 μA mm^−1^, 11.2±3.40 μS mm^−1^ and 27.0±7.44 cm^2^ V^−1^ s^−1^, respectively. The variation in the device performance has been widely observed in printed SWCNT transistors^11^ and could be improved by delicate control of the printing parameters^37^.

The device characteristics strongly depended on the density of the SWCNTs (see Supplementary Figs 6 and 7), which was controlled by the amount of SWCNT ink drop-casted on the substrates (Fig. 1e). Supplementary Figure 8 shows *I*_ON_/*W*, *g*_m_/*W*, *I*_ON/OFF_ and mobility as a function of density of nanotubes for *V*_D_=−2.0 V. As the density of nanotubes decreases from 25 to 6 tubes μm^−1^, the average of *I*_ON/OFF_ increases from 7,200 to 18,300. This increase of *I*_ON/OFF_ ratio at low nanotube density is possible due to the decrease in the probability of percolative transport through the metallic nanotubes^29^,^38^. As long as the percolation threshold is smaller than the density of CNTs but larger than that of the metallic tubes, all percolation paths from source to drain must involve at least one semiconducting segment. Gate modulation of this ‘week' connection of semiconducting SWCNTs ensures high *I*_ON/OFF_ ratio^39^,^40^. At high SWCNT densities, the density of metallic SWCNTs can exceed percolation threshold and the network forms continuous paths across the device, which results in high off current (*I*_OFF_) and low *I*_ON/OFF_. As a trade-off, low density of nanotubes leads to a decrease of *I*_ON_/*W* and *g*_m_/*W*. As a result of the direct dependence of mobility with *g*_m_, the average mobility also decreases from 27.0 to 9.22 cm^2^ V^−1^ s^−1^ (parallel model) with the decrease of the density of SWCNT from 25 to 6 tubes μm^−1^ (see Supplementary Note 5). In the present work, the SWCNT density for the TFT device was optimized to ∼25 tubes μm^−1^ to have a high average mobility of 27.0 cm^2^ V^−1^ s^−1^, and an average *I*_ON/OFF_ of 7,200, which meets the demand of an *I*_ON/OFF_ ratio of at least ∼10^3^–10^4^ for the application of digital logic circuits^28^,^39^.

**Figure 3:**
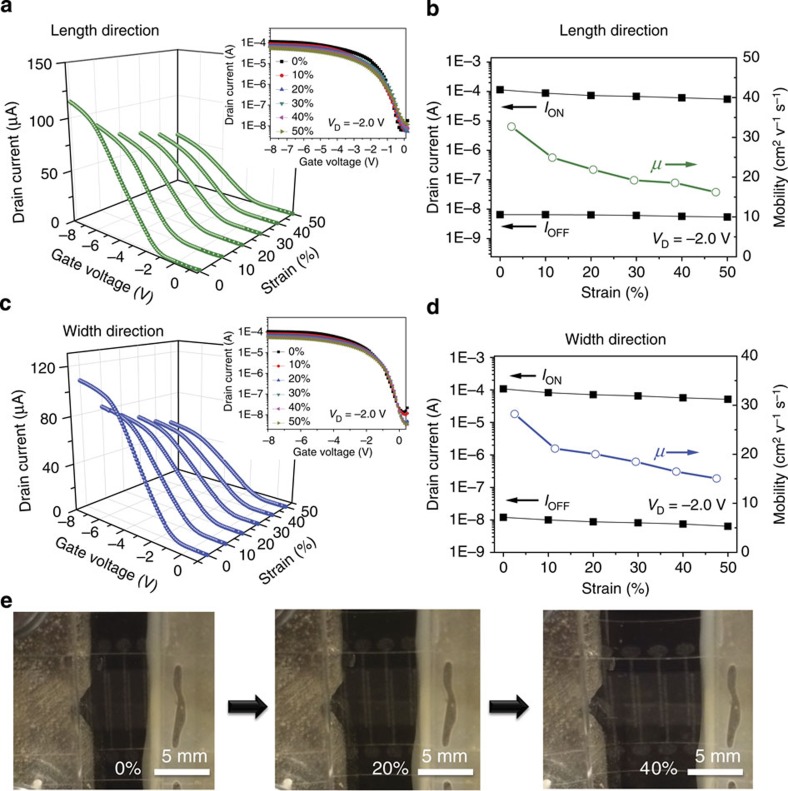
The device performance changes with tensile strain. (**a**) Typical transfer characteristics (*V*_D_=−2.0 V) of a TFT device under specific tensile strain applied along the channel length direction. The insets show log-scale characteristics. (**b**) *I*_ON,_ *I*_OFF_ and mobility as a function of applied strain along the channel length direction. (**c**) Typical transfer characteristics (*V*_D_=−2.0 V) of a device under specific tensile strain applied along the channel width direction. The insets show log-scale characteristics. (**d**) *I*_ON,_ *I*_OFF_ and mobility as a function of applied strain along the channel width direction. (**e**) Magnified photographs of a device at specified strains applied along the channel length direction. Scale bar=5 mm.

To investigate the electrical properties of the device under mechanical deformation, the transfer characteristics were further measured during the application of a uniaxial strain along either the channel length (Fig. 3a,b) or channel width (Fig. 3c,d). The I_ON_ value is declined by 52 and 51% with the strain increase from 0 to 50% along the channel length and width directions respectively (see Supplementary Fig. 9). In the meanwhile, *I*_OFF_ also exhibits a slight decrease under strain of up to 50%: the value is dropped by 17 and 46% with respect to the unstretched values along the channel length and width directions, respectively. The decrease in the source-drain channel current during stretching is mainly attributed to the increase in the contact resistance between the SWCNTs when stretched (see Supplementary Fig. 10)^19^,^41^,^42^. The mobility determined from the *g*_m_ and capacitance data (see Supplementary Fig. 11) exhibits a decrease, from 32.7 and 28.2 cm^2^ V^−1^ s^−1^ before stretching, to 16.2 and 15.0 cm^2^ V^−1^ s^−1^ at 50% strain along channel length and width directions, respectively. It is suggested that the decrease in capacitance of EDC with increasing strain is the main cause leading to the decrease of mobility (see Supplementary Fig. 11). The chain mobility of PEG in the DEC would be restricted when tensile strain is applied on the EDC, which results in the decrease of dielectric constant (see Supplementary Note 6). The elongation of the channel length between the AgNW-PUA source and drain electrodes can be obviously observed from the magnified photographs of TFTs at specific strain applied along channel length direction (Fig. 3e). The device fails when the strain is over 50% because of the tearing of elastomeric dielectric. Furthermore, *I*_ON_/*W*, *g*_m_/*W* and I_ON/OFF_ at strain of 50% along the channel length and width directions, which can be obtained from the transfer characteristics from Fig. 3a–c, are calculated to be 11.0 μA mm^−1^, 8.22 μS mm^−1^ and 8,400 along the channel length direction, and 10.4 μA mm^−1^, 7.61 μS mm^−1^ and 15,400 along the channel width direction, respectively. It is of noteworthy that the electrical performance of the stretchable TFTs at 50% strain is still comparable to most unstretchable TFT devices using SWCNTs as the channel material.

**Figure 4:**
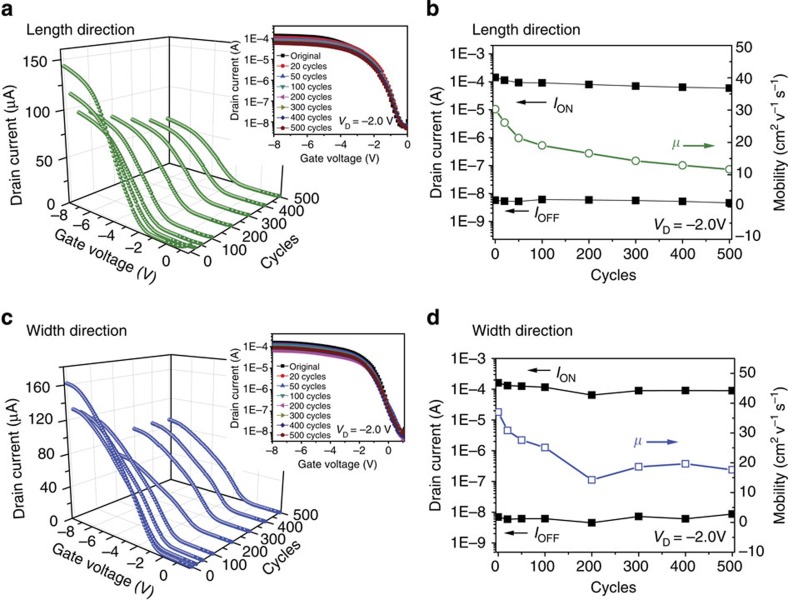
Fatigue testing when stretching and releasing 500 times. (**a**) Typical transfer characteristics (*V*_D_=−2.0 V) of a TFT device after specified cycles of 20% tensile strain applied along the channel length direction. The insets show log-scale characteristics. (**b**) Plots of *I*_ON_ and *I*_OFF_ at 0% strain during 500 cycles of continuous stretching–relaxing between 0 and 20% strains along the channel length direction. (**c**) Typical transfer characteristics (*V*_D_=−2.0 V) of a device after specified cycles of 20% tensile strain applied along the channel width direction. The insets show log-scale characteristics. (**d**) Plots of *I*_ON_ and *I*_OFF_ at 0% strain during 500 cycles of continuous stretching–relaxing between 0 and 20% strains along the channel width direction.

A fatigue test was subsequently performed by subjecting the TFT to 500 cycles of continuous stretch–relaxation between 0 and 20% strains along channel length or width direction. Figure 4 shows the electrical transfer characteristics, *I*_ON_ and *I*_OFF_ change during the continuous stretch–relaxation cycles. The *I*_ON_ for both strain direction decreased lightly over the 500 stretch–relaxation cycles, and the *I*_ON_ value remained fairly high value of ∼60 μA (initial value ∼140 μA) along channel length direction and ∼90 μA (initial value ∼160 μA) along channel width direction after 500 cycles (see Supplementary Fig. 9). In contrast, the *I*_OFF_ values remain relative constant throughout the cycling measurements along either direction. The calculated *I*_ON/OFF_ after 500 stretching–relaxing cycles along the channel length and width directions is ∼10,000 and 13,000, respectively. The mobility degraded relatively fast in the initial 200 cycles for both direction, but slowed down in the following 300 cycles. Mobility of 11.7 and 17.6 cm^2^ V^−1^ s^−1^ were obtained from the transfer characteristics curves after 500 stretch–relaxation cycles along channel length and width direction respectively. The retention of the device mobility after repeated strain cycles is still much higher than data reported in literature for all-printed SWCNT-based transistors^37^.

**Figure 5:**
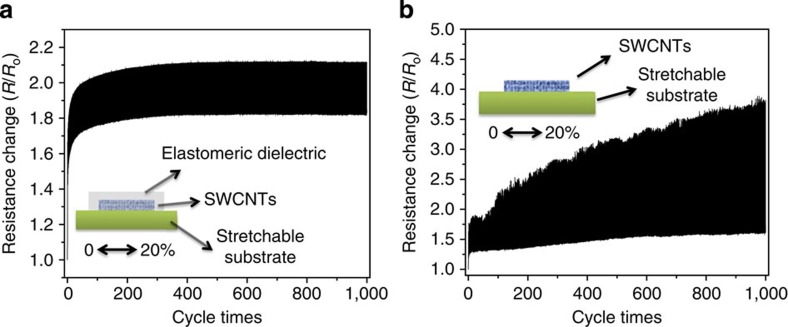
Mechanical compliance of SWCNT network on PUA substrate. Normalized transient resistance of SWCNT coated on PUA substrate with (**a**) and without (**b**) the elastomeric dielectric overcoat during 1,000 cycles of tensile stretching and releasing between 0 and 20% strains.

We speculate this good device performance for the device after subjecting repeated stretch–relaxation cycles is resulted from the unique structure where the EDC infiltrate into the SWCNT network and wraps around the nanotubes (Fig. 1a). The elastomeric dielectric wrapping around the SWCNT should constrain the junction disjoining and tube sliding, help distribute the stress more uniformly under tensile strain and thus increase the stretchability and stability of the SWCNT network under large strains^6^,^7^,^43^,^44^. Figure 5a,b display the normalized resistance (*R*/*R*_0_, the ratio of the instantaneous resistance at a specific tensile strain to the initial resistance at zero strain) for the SWCNT coated on PUA substrate with and without the EDC overcoat during 1,000 cycles of tensile stretching-releasing between 0 and 20% strains. It can be seen that after a gradually increase in the initial 100 cycles, the resistance of the SWCNT Network with EDC overcoat becomes very stable in the subsequent 900 cycles. The resistance increase during the first 100 cycles can be attributed to the alignment and sliding of the SWCNT network during deformation and the viscoelasticity of the elastomeric dielectric^6^,^44^. Overall, the elastomeric dielectric helps preserve the SWCNT network during large deformation very well. This trend of resistance change for the SWCNT network is consistent with the trend of mobility change for the TFT during continuous stretching–relaxing cycles as shown in Fig. 4b,d. In contrast, it can be observed from Fig. 5b that the SWCNT network without EDC overcoat exhibits a gradual increase in resistant continuously over 1,000 cycles.

### OLED driven by stretchable TFT

**Figure 6:**
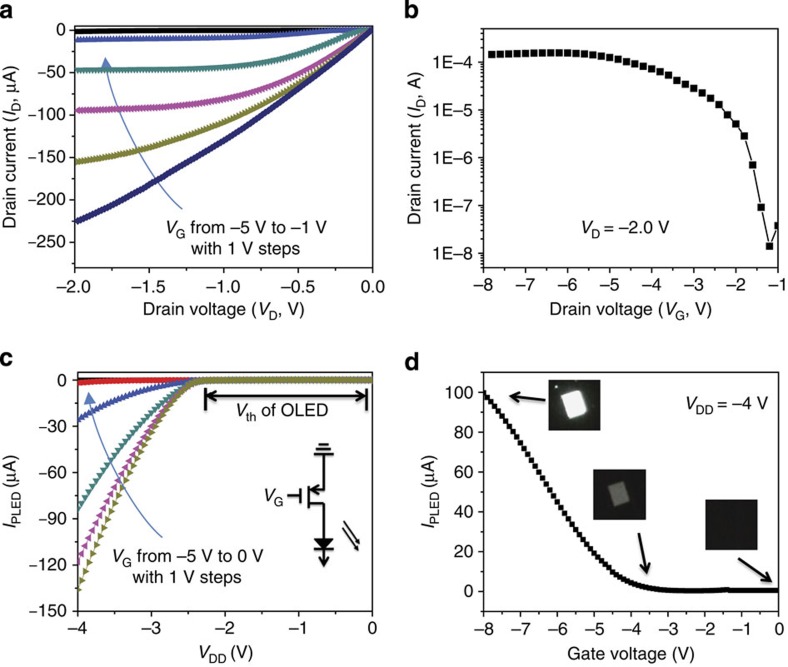
OLED control circuit driven by stretchable TFT. Stretchable SWCNT-AgNW TFT to control OLED devices. (**a**) Output (*I*_D_–*V*_D_) characteristics of the TFT device used to control an OLED with different gate voltage. (**b**) Transfer (*I*_D_–*V*_G_) characteristics of the TFT under *V*_D_=−2.0 V. (**c**) *I*_OLED_–*V*_DD_ characteristics of the OLED control circuit with different *V*_G_. Inset: schematic diagram of the OLED control circuit. (**d**) Plot of the *I*_OLED_ over *V*_G_ with *V*_DD_=−4.0 V. The inset photographs show the OLED brightness at specific *V*_G_.

To prove that the stretchable TFTs is applicable for stretchable active-matrix OLED displays, a prototype of white-light OLED driven by the stretchable SWCNT-AgNW TFT was demonstrated. Figure 6a,b present the output and transfer characteristics for the stretchable TFT used in the OLED control circuit. The TFT is able to source a current 150 μA at *V*_G_=−8 V and *V*_D_=−2.0 V with an *I*_ON/OFF_∼1.0 × 10^4^, which can meet the demand for driving and controlling the OLED. The white-light OLED used in this study has a sandwiched configuration of glass/ITO/PEDOT:PSS (40 nm)/white emissive polymer layer (75 nm)/CsF (1 nm)/Al (100 nm) (see Supplementary Note 7). In the drive circuit (inset of Fig. 6c), the connection was made by putting a probe onto the drain electrode of the device, and the probe is connected to a clamp by a conductive wire. The clamp is then fixed on the cathode of the OLED. A negative voltage was applied to anode of the OLED. Figure 6c displays a set of OLED current (*I*_OLED_) versus drain supply (*V*_DD_) characteristic curves for the OLED circuit under various input gate voltage *V*_G_. Good diode behaviour with a clear cutoff region and triode region could be clearly observed, indicating a good control from the stretchable TFT over the OLED. The cutoff voltage of *V*_DD_ is approximate −2.7 V, which is in accordance with the threshold voltage (*V*_th_, also called turn-on voltage) of the OLED (see Supplementary Fig. 12)^45^. Moreover, from Fig. 6d, the current flow through the OLED can be modulated by changing the input *V*_G_ by a factor of ∼1,000. This modulation could lead to the control of the OLED luminance. The inset optical photographs in Fig. 6d demonstrate the OLED operated with *V*_G_=−8 V (upper), −3 V (middle), 0 V (lower), respectively, obviously showing the OLED brightness modulation. When *V*_G_=−8 V, the OLED (with lighting area of 2.0 × 2.5 mm) is on and the light intensity is measured to be 218 cd m^−2^; when *V*_G_=0 V, the OLED is off and the luminance is measured to be lower than 0.01 cd m^−2^, the detection limit. Thus, the modulation for the OLED luminance is larger than 2 × 10^4^.

**Figure 7:**
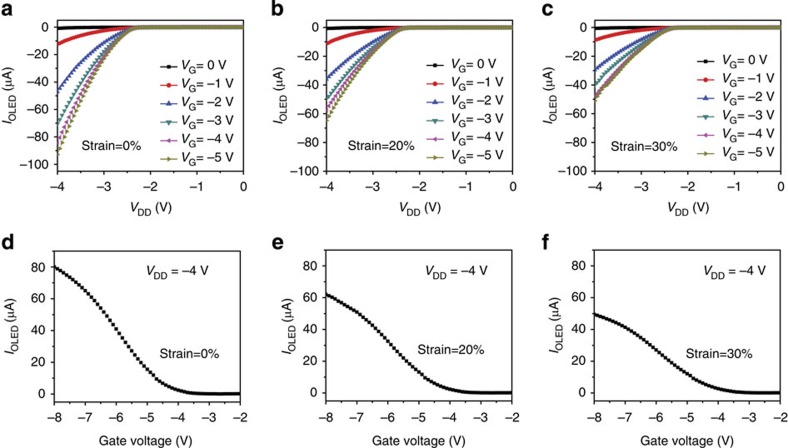
Characterization of OLED control circuit with stretchable TFT under different strains. Output (*I*_OLED_–*V*_DD_) characteristics of the OLED controlled by a stretchable SWCNT-AgNW TFT. The TFT is stretched along channel length direction by (**a**) 0%, (**b**) 20% and (**c**) 30% strains. The *V*_G_ is varied from 0 to −5.0 V in 1 V increments. Transfer (*I*_OLED_–*V*_G_) characteristics at *V*_DD_=−4.0 V for the TFT device used to control the OLED under 0% (**d**), 20% (**e**) and 30% (**f**) strains.

**Figure 8:**
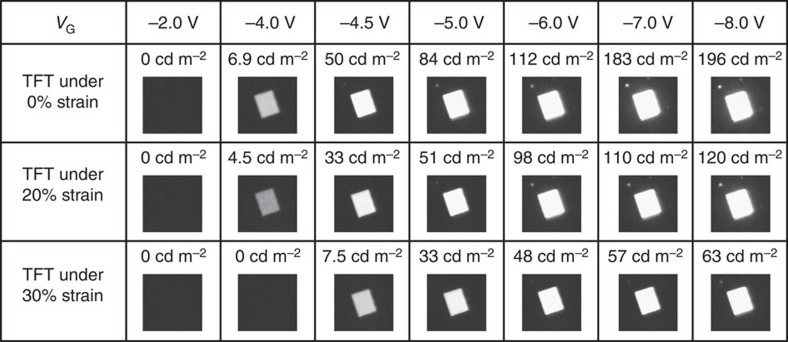
Luminance of an OLED driven by stretchable TFT under different inputs. Luminance was investigated for the TFT under strains (along channel length direction) of 0, 20 and 30%, respectively. *V*_DD_ is −4.0 V.

The OLED control circuit is further characterized by measuring the *I*_OLED_ with the stretchable TFT under 0, 20 and 30% strains applied along the channel length direction respectively. From the *I*_OLED_–*V*_DD_ characteristic in Fig. 7a–c, field-effect transistor-like behaviour with various curves corresponding to various values of input V_G_ is still clearly observed even when the TFT was stretched up to 20 and 30% strains respectively. The *I*_OLED_ flow through the OLED shows a mild decrease trend with the increasing strain. Figure 7d–f show the *I*_OLED_ versus *V*_G_ curves with the stretchable TFT under specific strains. A close examination of the curves reveals that the *V*_G_ modulation factor is ∼1,000, 750 and 650 for the TFT under strain of 0, 20 and 30%, respectively. The decrease of the modulation factor is attributed to the decrease of *I*_ON_, in consistency with the discussion above (Fig. 3). Figure 8 further shows the light-emitting properties of the OLED circuit at different *V*_G_ when *V*_DD_ is −4 V. The brightness of the OLED integrated with TFT under different strains of 0, 20 and 30% is shown for comparison. When the TFT was stretched to 20% strain, the brightness decreased from 196 cd m^−2^ (initial value at 0% strain) to 120 cd m^−2^ at *V*_G_=−8 V, which is still sufficient for display applications. When stretched up to 30%, the brightness declined by ∼68% to 63 cd m^−2^.

## Discussion

In summary, a high-performance, transparent and intrinsically elastomeric TFT have been fabricated using a solution-based printable process. The device is made of all stretchable materials, including AgNW-PUA composite conductor as the source, drain, and gate, a SWCNT network channel and PU-*co*-PEG elastomeric dielectric. The SWCNT network is infiltrated by the elastomeric dielectric, and wrapping the nanotubes without interrupting the nanotube–nanotube junction is beneficial for large-strain stretchability without significantly diminishing electric performance. The elastomeric TFT retains fairly high electrical performance at strains as high as 50% and can survive a large number of stretching cycles. The stretchable TFT is successfully adapted to switch a white-light OLED from totally dark to 196 cd m^−2^ or display brightness. The TFT layout and fabrication process could be adapted to fabricate a large of TFT to achieve stretchable active-matrix OLED display.

## Methods

### Materials

AgNWs were synthesized according to a reported procedure 46. Siliconized urethane acrylate oligomer (CN990), ethoxylated bisphenol A dimethacrylate (SR540), PEG (200) diacrylate (SR259) and methoxy PEG (350) monomethacrylate (SR550) were supplied by Sartomer. 2,2-Dimethoxy-2-phenylacetophenone (photoinitiator) was obtained from Sigma-Aldrich. Semiconducting SWCNT (99%+) was obtained from Nanointegris Inc. Fluorosurfactant FC-4430 was purchased from 3M.

### Preparation of AgNW-PUA composite source/drain and gate electrode

To pattern the source/drain on glass, a dispersion of AgNWs in methanol (0.2 wt%) was spray-printed on glass through a patterned shadow mask using Paasche air brush. To fabricate the gate electrode on glass, a dispersion of AgNW (0.5 wt%) in IPA was drop-casted into a thin line at one end of the substrate and drawn down with a Meyer rod to spread the solution to form a uniform coating. The resulting transparent conductive coating (both source/drain and gate) on the glass substrates was then coated with a precursor solution consisting of 100 weight parts CN990, 20 parts SR540 and 1 part photoinitiator. The coatings were cured under UV at 2.5 W cm^-2^ intensity and peeled off of the release substrate. The AgNW source/drain electrode with channel length of 100 μm and channel width of 5,000 μm and gate electrode were transferred as the conductive surface of the resulting transparent composite electrodes. Both the AgNW-PUA composite source/drain and gate electrode were prepared with sheet resistance controlled to be 15 Ω sq^−1^^8^.

### Preparation of elastomeric dielectric

The precursor of DEC was formulated by mixing the components CN990, SR259, SR550 and photoinitiator, in an optimized weight ratio of 1:1:1:0.015. CN990 was chosen for the high stretchability and good bonding of its homopolymer with the AgNW-PUA source/drain and gate composite electrode. SR259 and SR550 were chosen for the good flexibility and high dielectric constant of their homopolymer and low viscosity of their monomer (25 and 19 c.p.s. @ 25 ^o^C, respectively). SR550 can serve as a polar side chain to further improve the dielectric constant.

### Fabrication of fully transparent and stretchable transistor

The device fabrication started with printing SWCNT on the AgNW-PUA composite source/drain electrode. First, SWCNT ink was prepared by mixing 99%+ Semiconducting -SWCNT aqueous solutions (used as-received from Nanointegris Inc.), DI-water, propylene glycol and Fluorosurfactant at the ratio of 25:5:5:1, followed by untrasonicating for 20 min. Subsequently, a certain amount of SWCNT ink was uniformly drop-casted on the conductive surface of AgNW-PUA source/drain composite electrode, followed by heating up to 90 ^o^C on a hot plate to dry the SWCNT network. SWCNT densities on the source/drain channel was controlled by the amount of SWCNT ink being 6, 15 and 30 μl on 0.5 × 1.5 cm^2^ AgNW-PUA composite source/drain electrode to obtain SWCNT densities of ∼6, ∼13 and ∼25 tubes μm^−1^, respectively. The SWCNT/AgNW-PUA source/drain was rinsed with Deionized water and IPA alternatively to remove the sodium dodecyl sulphate surfactant and Fluorosurfactant, followed by drying in vacuum for 1 h. SWCNT was patterned by O_2_ plasma etching to the source/drain electrode. Then, the elastomeric dielectric precursor was overcoated on the SWCNT/AgNW-PUA source/drain substrate, spin-printed at 8,000 r.p.m. for 1 min and then UV-cured in nitrogen gas. Transparent tape (3M) was used to define the openings for the source/drain electrode and peeled off after polymerizing the elastomeric dielectric. Finally, the resulting elastomeric dielectric/SWCNT/AgNW-PUA source/drain structure was laminated with another 15 Ω sq^−1^ AgNW-PUA composite electrode as gate on top of the dielectric layer to complete the device fabrication.

### Characterization method

Cyclic strain tests were performed on a motorized linear stage with built-in controller (Zaber Technologies Inc.). A Keithley 2000 digital multimeter was used tomonitor the resistance change. Strain and resistance data were recorded with a custom-made LabViewcode. The transmittance spectra were recorded utilizing a Shimadzu UV-1700 spectrophotometer. SEM was performed on a JEOL JSM-6701F scanning electron microscope. The dielectric constant and capacitance of the elastomeric dielectric were measured using LCR meter (GW INSTEK LCR-819.) the by fabricating 12 ITO–elastomeric dielectric–evaporated alumina capacitors and averaging the unit area capacitance of all 12 capacitors. Kelvin probe force microscopy was conducted with a Bruker Dimension Icon Scanning Probe Microscope to measure the work function of AgNW. Transistor electrical characterization was performed with two Keithley 2400 source meter. The measurement sequences were controlled by a custom-made LabViewcode. All the transistor measurements were tested under ambient atmospheric conditions.

## Additional information

**How to cite this article:** Liang, J. *et al*. Intrinsically-stretchable and transparent thin-film transistors based on printable silver nanowires, carbon nanotubes and an elastomeric dielectric. *Nat. Commun.* 6:7647 doi: 10.1038/ncomms8647 (2015).

## Supplementary Material

Supplementary Information Supplementary Figures 1-12, Supplementary Table 1, Supplementary Notes 1-7 and Supplementary References

## References

[b1] White M. S. . Ultrathin, highly flexible and stretchable PLEDs. Nat. Photonics 7, 811–816 (2013).

[b2] Xu S. . Soft microfluidic assemblies of sensors, circuits, and radios for the skin. Science 344, 70–74 (2014). 24700852 10.1126/science.1250169

[b3] Savagatrup S., Printz A. D., O'Connor T. F., Zaretski A. V. & Lipomi D. J. Molecularly stretchable electronics. Chem. Mater. 26, 3028–3041 (2014).

[b4] Kim R. H. . Waterproof AlInGaP optoelectronics on stretchable substrates with applications in biomedicine and robotics. Nat. Mater. 9, 929–937 (2010). 20953185 10.1038/nmat2879

[b5] Filiatrault H. L., Porteous G. C., Stephen R., Davidson G. J. E. & Breen T. Stretchable light-emitting electrochemical cells using an elastomeric emmissive material. Adv. Mater. 24, 2673–2678 (2012). 22451224 10.1002/adma.201200448

[b6] Yu Z., Niu X., Liu Z. & Pei Q. Intrinsically stretchable polymer light-emitting devices using carbon nanotube-polymer composite electrodes. Adv. Mater. 23, 3989–3994 (2011). 21796688 10.1002/adma.201101986

[b7] Liang J. . Silver nanowire percolation network soldered with graphene oxide at room temperature and its application for fully stretchable polymer light-emitting diodes. ACS Nano 8, 1590–1600 (2014). 24471886 10.1021/nn405887k

[b8] Liang J., Li L., Niu X., Yu Z. & Pei Q. Elastomeric polymer light-emitting devices and displays. Nat. Photonics 7, 817–824 (2013).

[b9] Zhang J., Wang C. & Zhou C. Rigid/flexible transparent electronics based on separated carbon nanotube thin-film transistors and their application in display electronics. ACS Nano 6, 7412–7419 (2012). 22788112 10.1021/nn3026172

[b10] Kim S., Kim S., Park J., Ju S. & Mohammadi S. Fully transparent pixel circuits driven by random network carbon nanotube transistor circuitry. ACS Nano 4, 2994–2998 (2010). 20450163 10.1021/nn1006094

[b11] Lau P. H. . Fully printed, high performance carbon nanotube thin-film transistors on flexible substrates. Nano Lett. 13, 3864–3869 (2013). 23899052 10.1021/nl401934a

[b12] Chen P. C. . Fully printed separated carbon nanotube thin film transistor circuits and its application in organic light emitting diode control. Nano Lett. 11, 5301–5308 (2011). 22050730 10.1021/nl202765b

[b13] Vosgueritchian M., Tok J. B. H. & Bao Z. Stretchable LEDs: Light-emitting electronic skin. Nat. Photonics 7, 769–771 (2013).

[b14] Kim D. H., Xiao J. L., Song J. Z., Huang Y. G. & Rogers J. A. Stretchable, curvilinear electronics based on inorganic materials. Adv. Mater. 22, 2108–2124 (2010). 20564250 10.1002/adma.200902927

[b15] Khang Y. D., Rogers J. A. & Lee H. H. Mechanical buckling: mechanics, metrology, and stretchable electronics. Adv. Funct. Mater. 19, 1526–1536 (2009).

[b16] Rogers J. A., Someya T. & Huang Y. G. Materials and mechanics for stretchable electronics. Science 327, 1603–1607 (2010). 20339064 10.1126/science.1182383

[b17] Kim D. H. . Materials and noncoplanar mesh designs for integrated circuits with linear elastic responses to extreme mechanical deformations. Proc. Natl. Acad. Sci. USA 105, 18675–18680 (2008). 19015528 10.1073/pnas.0807476105 PMC2584145

[b18] Kaltenbrunner M. . An ultra-lightweight design for imperceptible plastic electronics. Nature 499, 458–463 (2013). 23887430 10.1038/nature12314

[b19] Chae S. H. . Transferred wrinkled Al2O3 for highly stretchable and transparent graphene-carbon nanotube transistors. Nat. Mater. 12, 403–409 (2013). 23455851 10.1038/nmat3572

[b20] Xu F. . Highly stretchable carbon nanotube transistors with ion gel gate dielectrics. Nano Lett. 14, 682–686 (2014). 24382263 10.1021/nl403941a

[b21] Park K. . Stretchable, transparent zinc oxide thin film transistors. Adv. Funct. Mater. 20, 3577–3582 (2010).

[b22] Chortos A. . Highly stretchable transistors using a microcracked organic semiconductor. Adv. Mater. 26, 4253–4259 (2014). 24740928 10.1002/adma.201305462

[b23] Pu J. . Fabrication of stretchable MoS2 thin-film transistors using elastic ion-gel gate dielectrics. Appl. Phys. Lett. 103, 023505 (2013).

[b24] Lee S. . Stretchable graphene transistors with printed dielectrics and gate electrodes. Nano Lett. 11, 4642–4646 (2011). 21973013 10.1021/nl202134z

[b25] Shin M. . Highly stretchable polymer transistors consisting entirely of stretchable device components. Adv. Mater. 26, 3706–3711 (2014). 24664816 10.1002/adma.201400009

[b26] Liu H. L. . A new kind of electro-active polymer composite composed of silicone elastomer and polyethylene glycol. J. Phys. D Appl. Phys 45, 485303 (2012).

[b27] Mali C. S., Chavan S. D., Kanse K. S., Kumbharkhane A. C. & Mehrotra S. C. Dielectric relaxation of poly ethylene glycol–water mixtures using time domain technique. Indian J. Pure Appl. Phys. 45, 476–481 (2007).

[b28] Wang C. . Extremely bendable, high-performance integrated circuits using semiconducting carbon nanotube networks for digital, analog, and radio-frequency applications. Nano Lett. 12, 1527–1533 (2012). 22313389 10.1021/nl2043375

[b29] Wang C. . Wafer-scale fabrication of separated carbon nanotube thin-film transistors for display applications. Nano Lett. 9, 4285–4291 (2009). 19902962 10.1021/nl902522f

[b30] Xu H., Chen L., Hu L. B. & Zhitenev N. Contact resistance of flexible, transparent carbon nanotube films with metals. Appl. Phys. Lett. 97, 143116 (2010).

[b31] Jackson R. & Graham S. Specific contact resistance at metal/carbon nanotube interfaces. Appl. Phys. Lett. 94, 012109 (2009).

[b32] Cao Q. & Rogers J. A. Random networks and aligned arrays of single-walled carbon nanotubes for electronic device applications. Nano Res 1, 259–272 (2008).

[b33] Kim W. . Hysteresis caused by water molecules in carbon nanotube field-effect transistors. Nano Lett. 3, 193–198 (2003).

[b34] Jin S. H. . Sources of hysteresis in carbon nanotube field-effect transistors and their elimination via methylsiloxane encapsulants and optimized growth procedures. Adv. Funct. Mater. 22, 2276–2284 (2012).

[b35] Cao Q. . Gate capacitance coupling of singled-walled carbon nanotube thin-film transistors. Appl. Phys. Lett. 90, 023516 (2007).

[b36] Kang S. J. . High-performance electronics using dense, perfectly aligned arrays of single-walled carbon nanotubes. Nat. Nanotechnol 2, 230–236 (2007). 18654268 10.1038/nnano.2007.77

[b37] Shi J., Guo C. X., Chan-Park M. B. & Li C. M. All-printed carbon nanotube finFETs on plastic substrates for high-performance flexible electronics. Adv. Mater. 24, 358–361 (2012). 22162001 10.1002/adma.201103674

[b38] Park S., Vosguerichian M. & Bao Z. N. A review of fabrication and applications of carbon nanotube film-based flexible electronics. Nanoscale 5, 1727–1752 (2013). 23381727 10.1039/c3nr33560g

[b39] Pimparkar N., Cao Q., Rogers J. A. & Alam M. A. Theory and practice of “striping” for improved ON/OFF ratio in carbon nanonet thin film transistors. Nano Res 2, 167–175 (2009).

[b40] Snow E. S., Novak J. P., Campbell P. M. & Park D. Random networks of carbon nanotubes as an electronic material. Appl. Phys. Lett. 82, 2145–2147 (2003).

[b41] Li Q. . Structure-dependent electrical properties of carbon nanotube fibers. Adv. Mater. 19, 3358–3363 (2007).

[b42] Zhang Y. . Polymer-embedded carbon nanotube ribbons for stretchable conductors. Adv. Mater. 22, 3027–3031 (2010). 20518036 10.1002/adma.200904426

[b43] Xu F. & Zhu Y. Highly conductive and stretchable silver nanowire conductors. Adv. Mater. 24, 5117–5122 (2012). 22786752 10.1002/adma.201201886

[b44] Hu W., Niu X., Zhao R. & Pei Q. Elastomeric transparent capacitive sensors based on an interpenetrating composite of silver nanowires and polyurethane. Appl. Phys. Lett. 102, 083303 (2013).

[b45] Li L. . A solution processed flexible nanocomposite electrode with efficient light extraction for organic light emitting diodes. Sci. Rep 4, 4307 (2014). 24632742 10.1038/srep04307 PMC3955904

[b46] Sun Y., Gates B., Mayers B. & Xia Y. Crystalline silver nanowires by soft solution processing. Nano Lett. 2, 165–168 (2002).

