# Supplementary material for: Intrinsically stretchable and transparent thin-film transistors based on printable silver nanowires, carbon nanotubes and an elastomeric dielectric
Source: Nat Commun. 2015 Jul 15;6:7647. doi: 10.1038/ncomms8647 (PMC4518309; doi:10.1038/ncomms8647)
Supplement: Supplementary Information — Supplementary Figures 1-12, Supplementary Table 1, Supplementary Notes 1-7 and Supplementary References [file ncomms8647-s1.pdf]

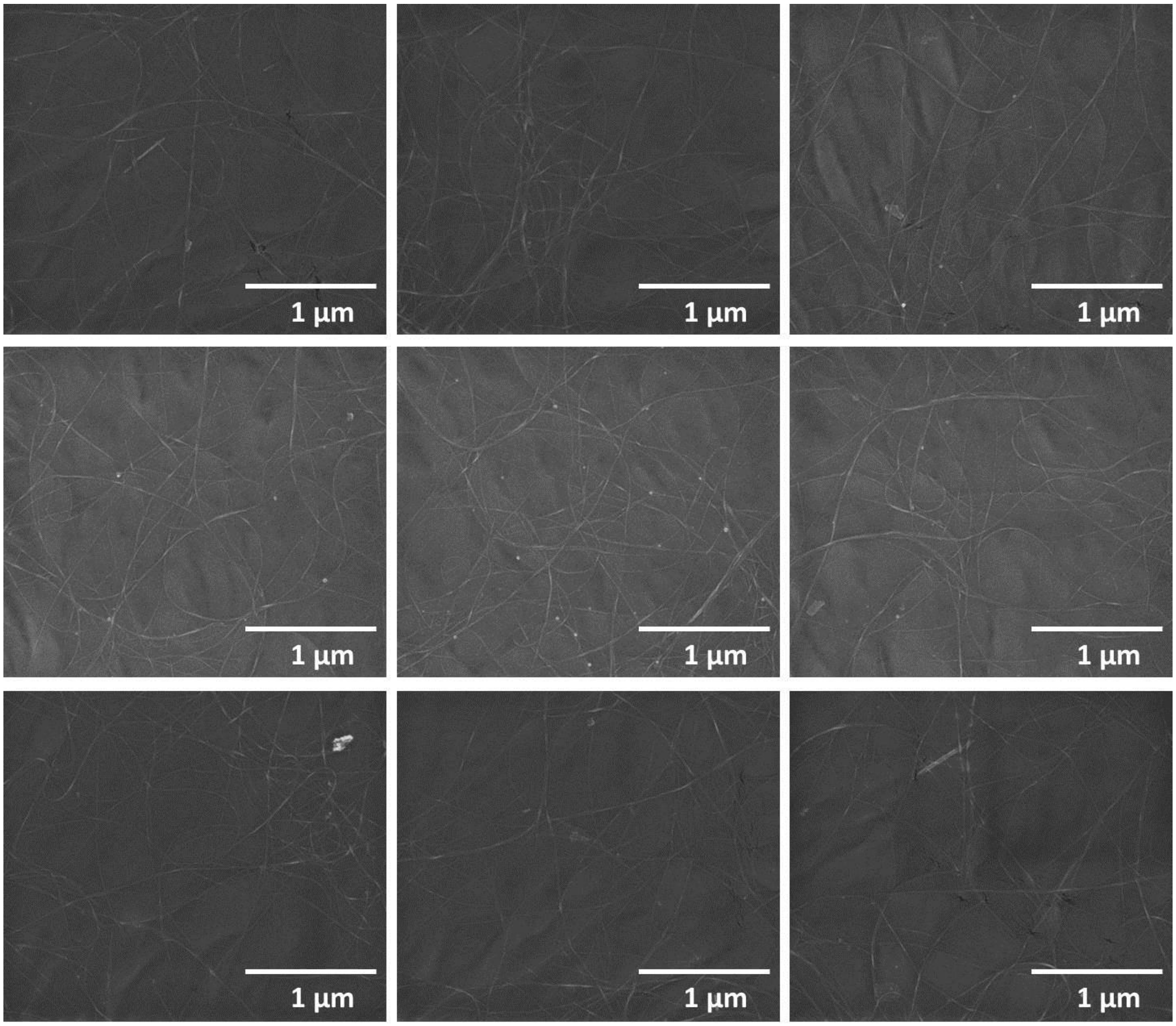

Supplementary Figure 1. SEM images of SWCNTs printed at 9 different locations on the AgNW-PUA composite source/drain electrodes with 15 µl deposition amount of SWCNT ink (99%+ Semiconducting-SWCNT aqueous solutions (Nanointegris Inc), Deionic water, propylene glycol and Fluorosurfactant at the ratio of 25:5:5:1). The SWCNT networks are provided with a tube density about 10-15 tubes μm^{-1} with an average value ~13 tubes μm^{-1}, indicating a uniform deposition of SWCNTs on the substrate.

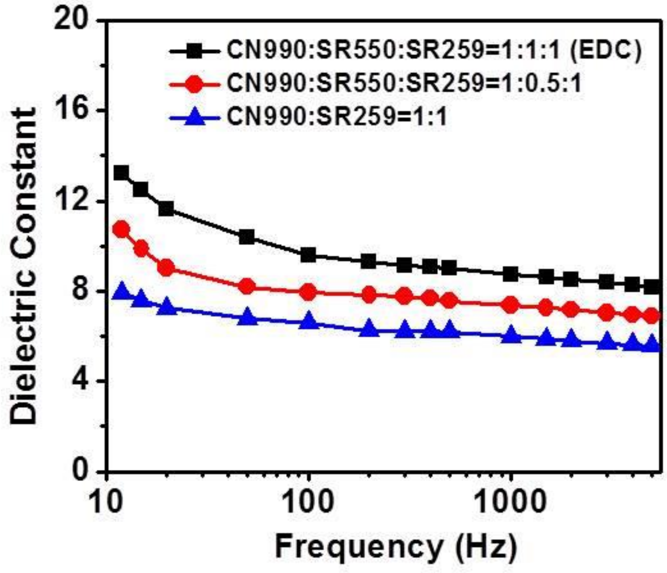

Supplementary Figure 2. Dielectric constant of copolymer at various ratios of CN990, SR550 and SR259.

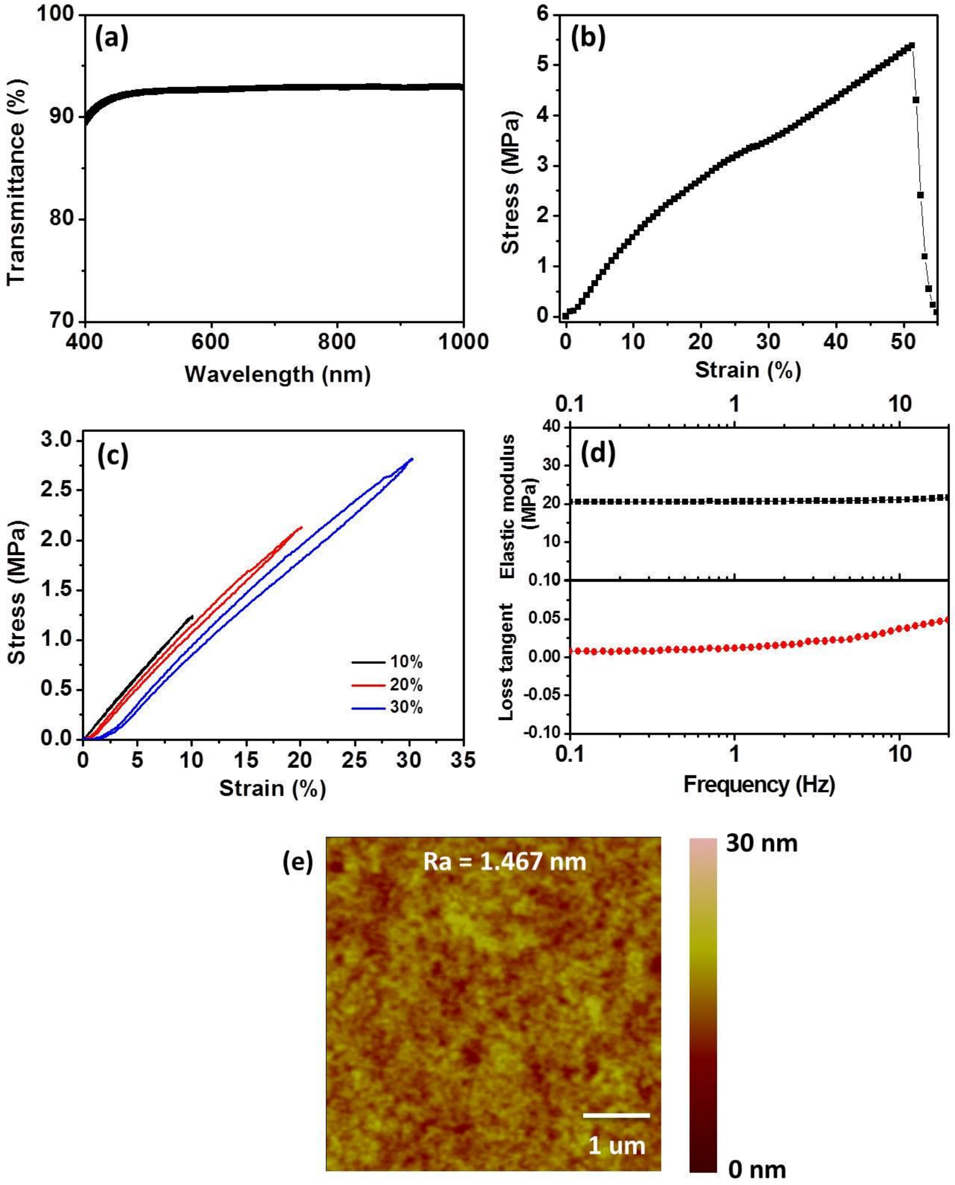

Supplementary Figure 3. (a) Transmittance of the EDC film with thickness of 150 μm. (b) Stress-strain responses of the EDC film. (c) The fifth cycle of loading and unloading (up to 10, 20, and 30% strain) for the EDC. (d) Elastic modulus and mechanical loss tangent vs frequency for the EDC. A 1% offset strain to prevent buckling and an oscillatory strain of 0.1% was applied. (e) AFM image of EDC on AgNW-PUA composite source/drain electrodes. The scanned area is 5 × 5 μm^2.

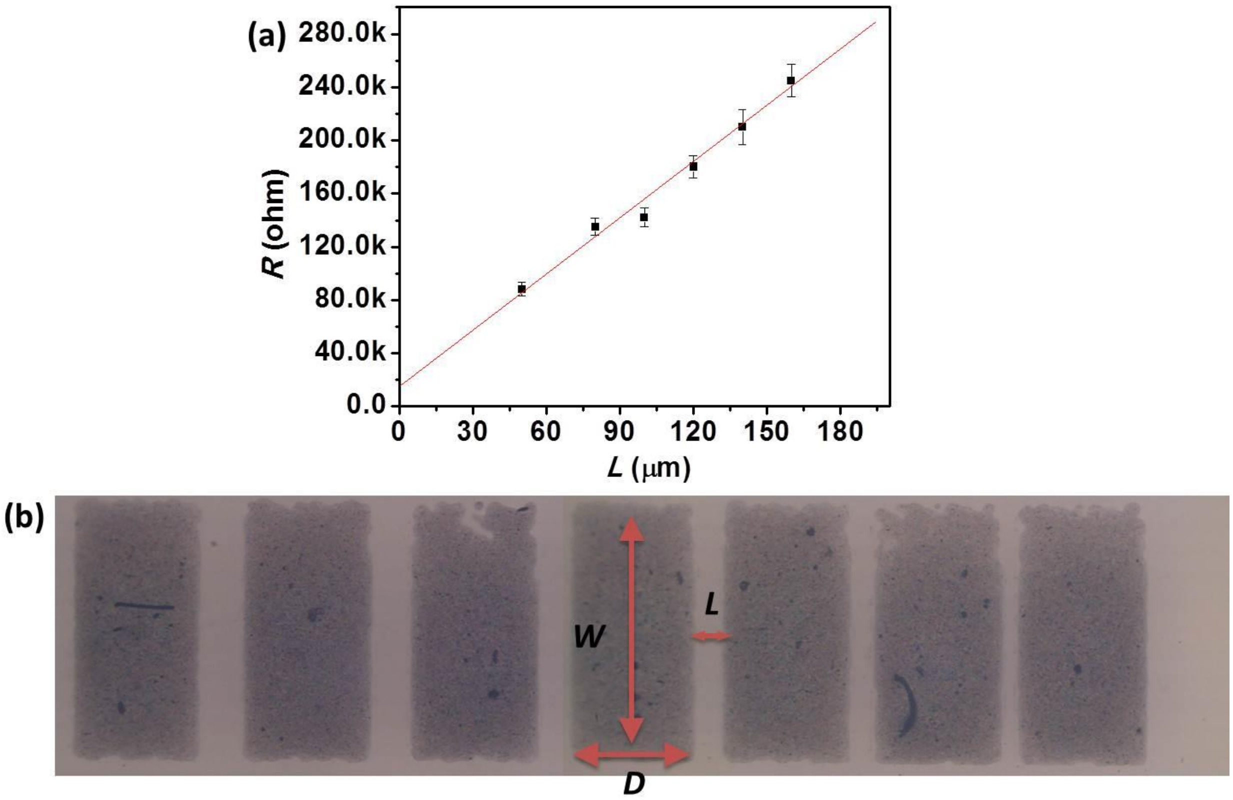

Supplementary Figure 4. (a) Measured resistance plotted versus different spacings for AgNW electrode contacts. *D* = 500 µm, and *W* = 1,000 µm. (b) Optical image of a TLM test structure of AgNW electrode contacts..

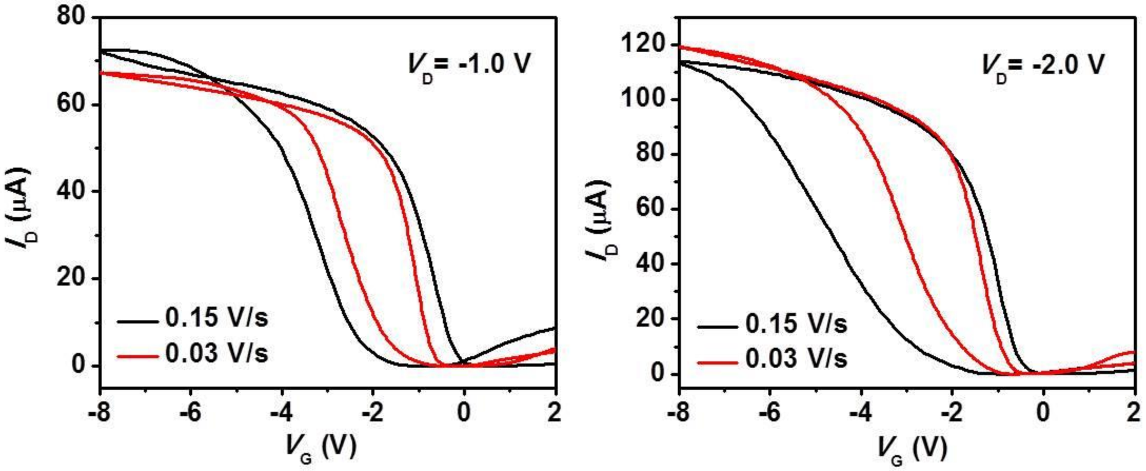

Supplementary Figure 5. I_D-V_G characteristics of devices at various sweep rates (0.15 and 0.03 V s^{-1}) with (a) V_D= -1.0 V and (b) V_D = -2.0 V.

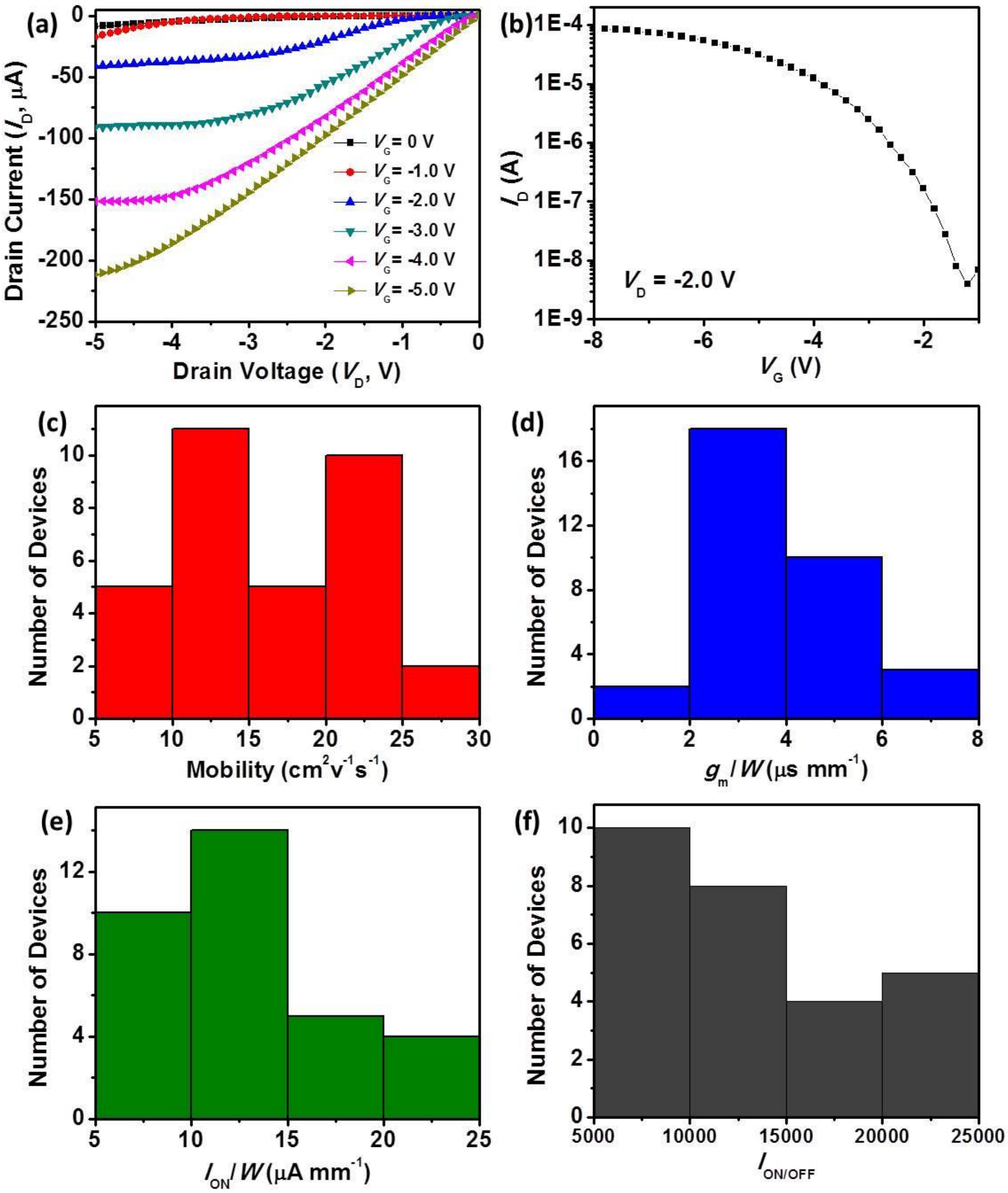

Supplementary Figure 6. Output characteristics of a typical SWCNT-AgNW TFT (L = 100 μm and W = 5,000 μm) with a density of SWCNT about 13 tubes μm^{-1} with. V_G from 0 to -5 V in 1 V steps. (b) Transfer characteristics of the same device with V_D from -1.0 to -3.0 in 1.0 step. (d)-(g) Histograms of the TFTs showing the statistical distribution of (d) Mobility, (e) g_m/W, (f) I_{ON}/W, and (g) $I_{ON/OFF}$.

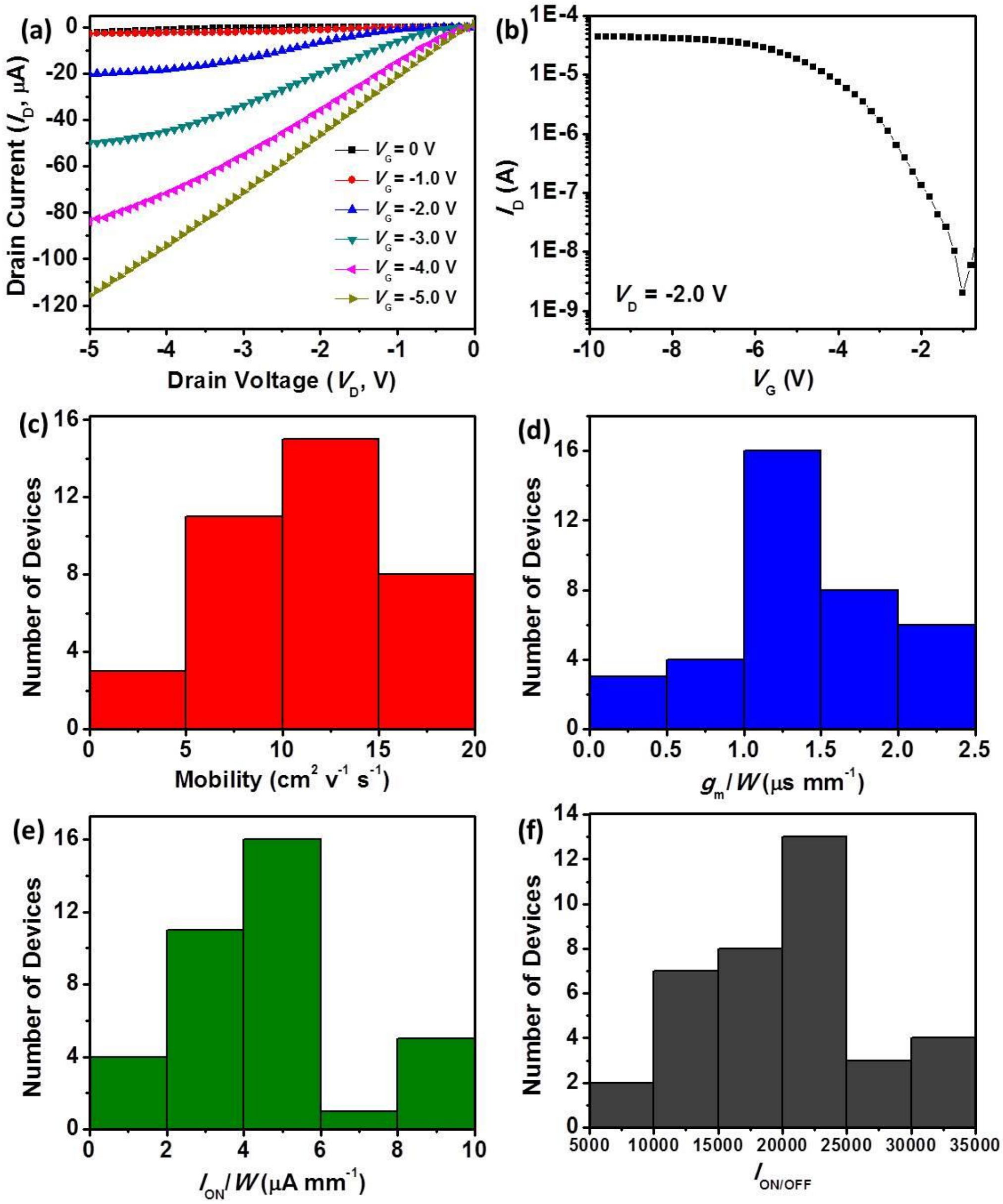

Supplementary Figure 7. Output characteristics of a typical SWCNT-AgNW TFT (L = 100 μm and W = 5,000 μm) with a density of SWCNT about 6.0 tubes μm^{-1} with. V_G from 0 to -5 V in 1 V steps. (b) Transfer characteristics of the same device with V_D from -1.0 to -3.0 in 1.0 step. (d)-(g) Histograms of the TFTs showing the statistical distribution of (d) Mobility, (e) g_m/W, (f) I_{ON}/W, and (g) $I_{ON/OFF}$.

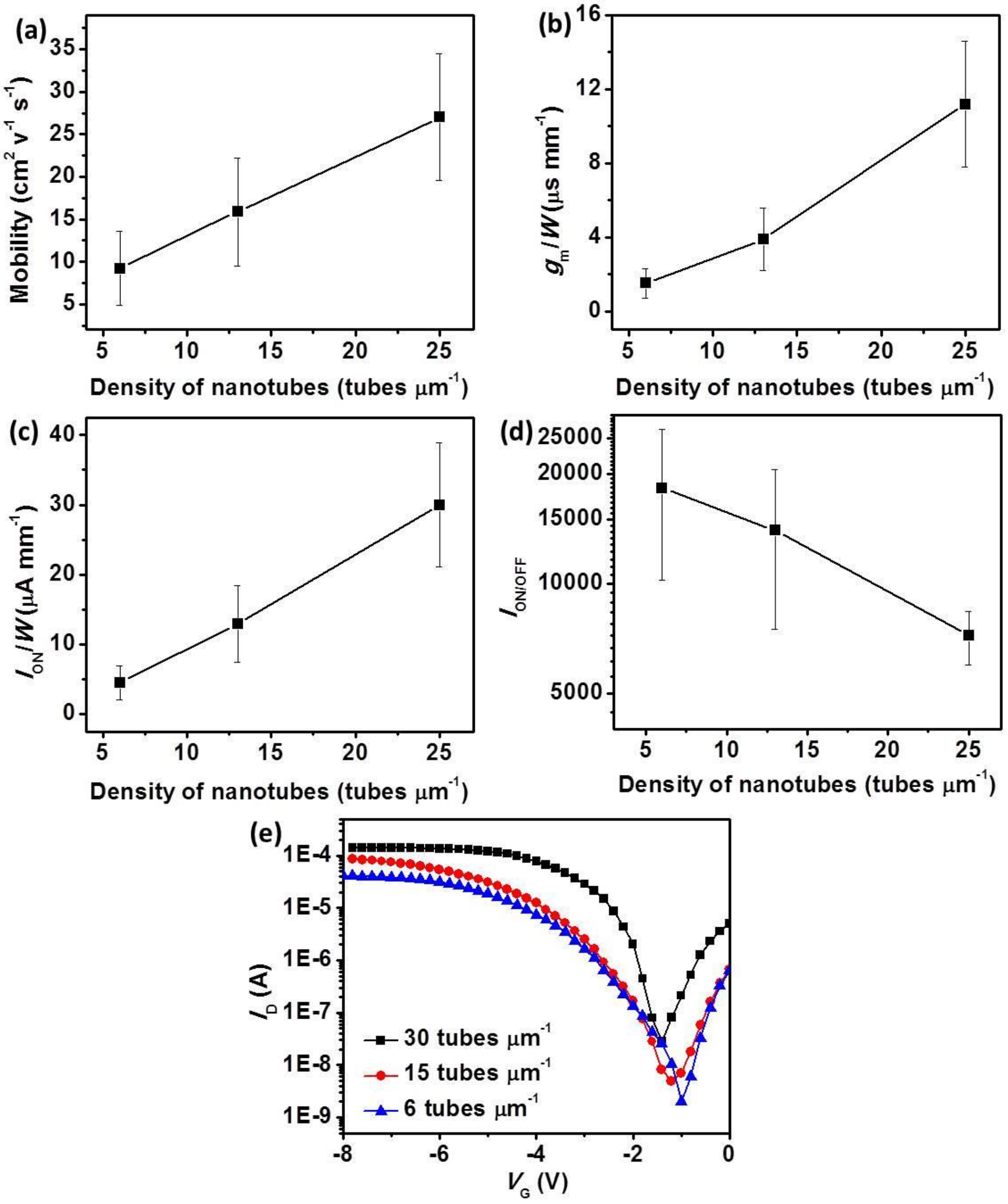

Supplementary Figure 8. (a) The mobility, (b) gm/W, (c) I_{ON}/W, and (d) $I_{ON/OFF}$ in terms of the SWCNT density for the SWCNT-AgNW TFTs for V_D= -2.0 V. (e) Transfer characteristics of devices with different SWNT densities at V_D = -2.0 V.

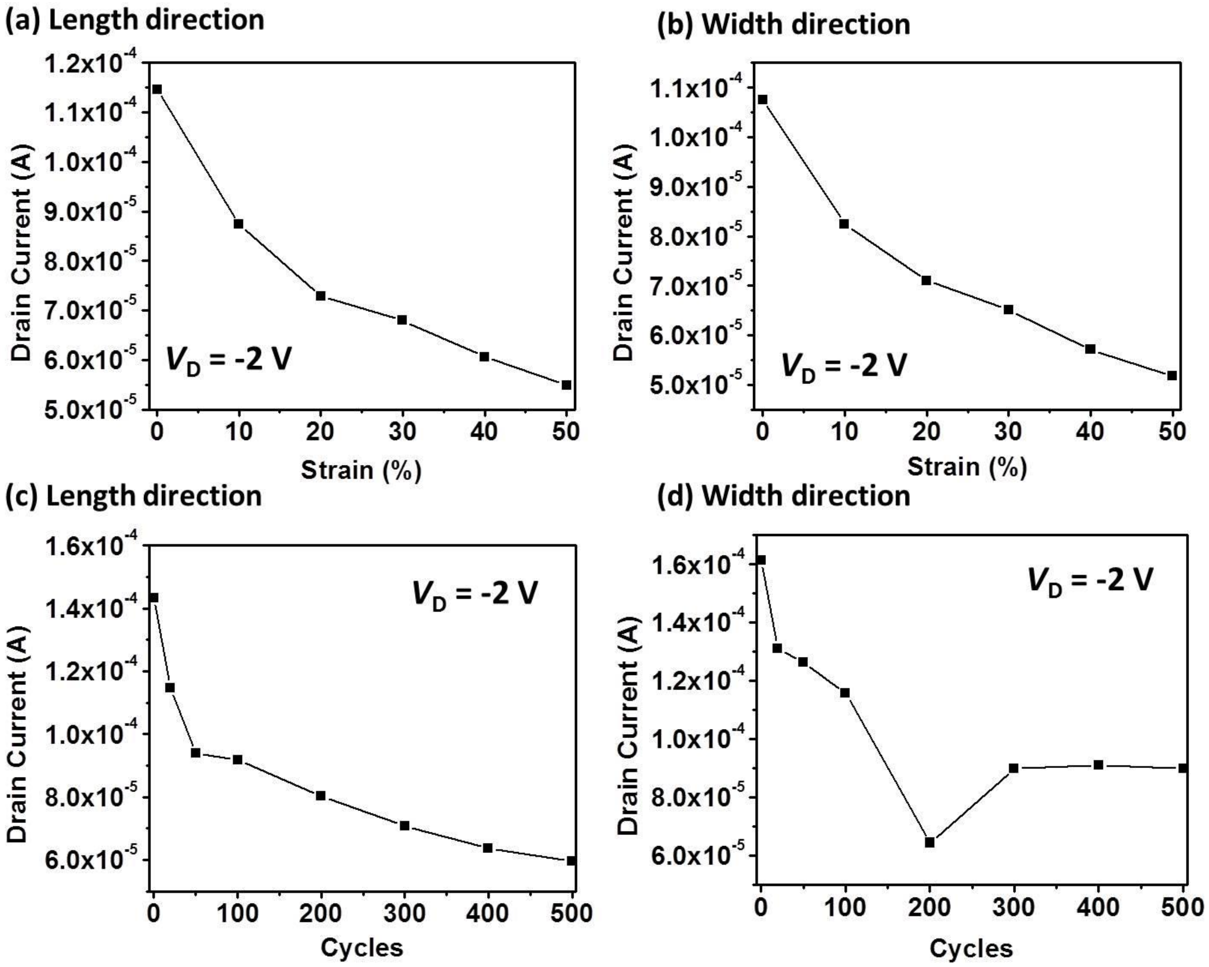

Supplementary Figure 9. I_{ON} as a function of applied strain along the channel length (a), and width (b) direction on linear scale. Plots of I_{ON} at 0% strain during 500 cycles of continuous stretching-relaxing between 0% and 20% strains along the channel length (c) and width (d) direction on linear scale.

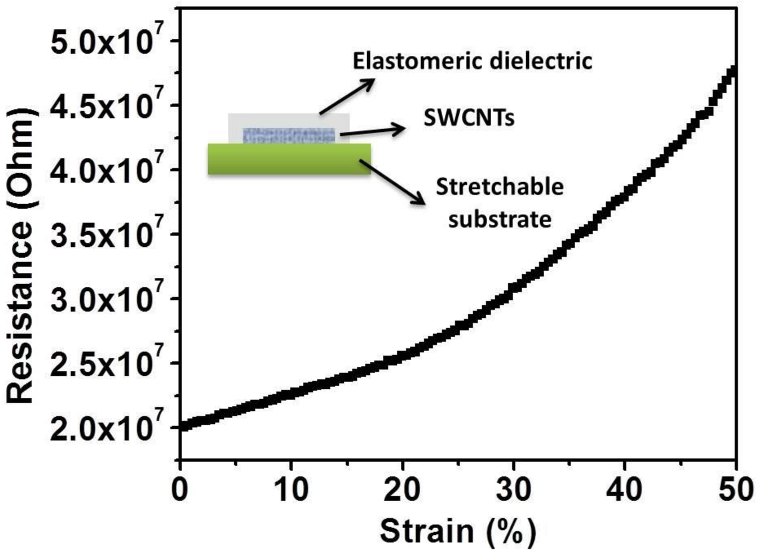

Supplementary Figure 10. Resistance change of the SWCNT network coated on PUA substrate with EDC overcoat with increasing strain.

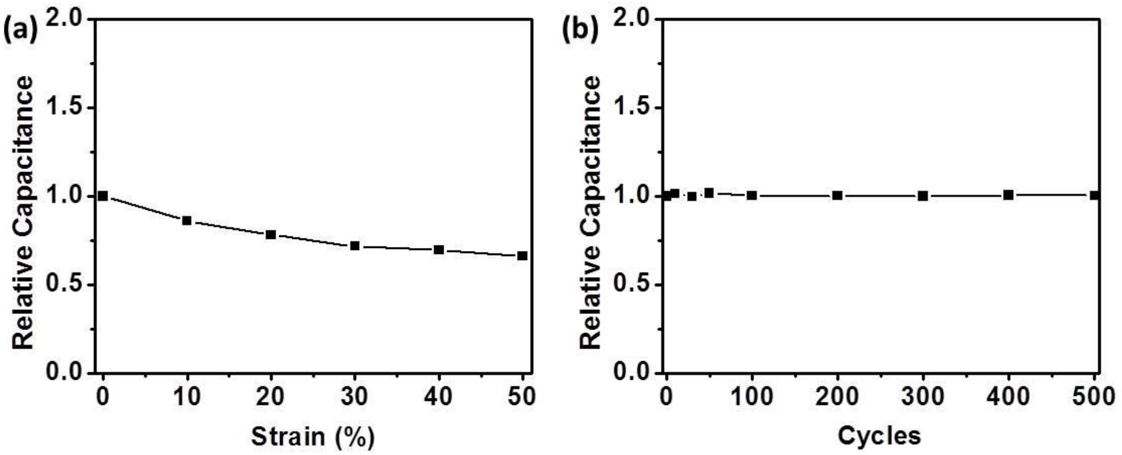

Supplementary Figure 11. Properties of the EDC with strain. (a) Relative capacitance as a function of strains. (b) Relative capacitance as a function of continuous stretching-relaxing cycles between 0% and 20% strains.

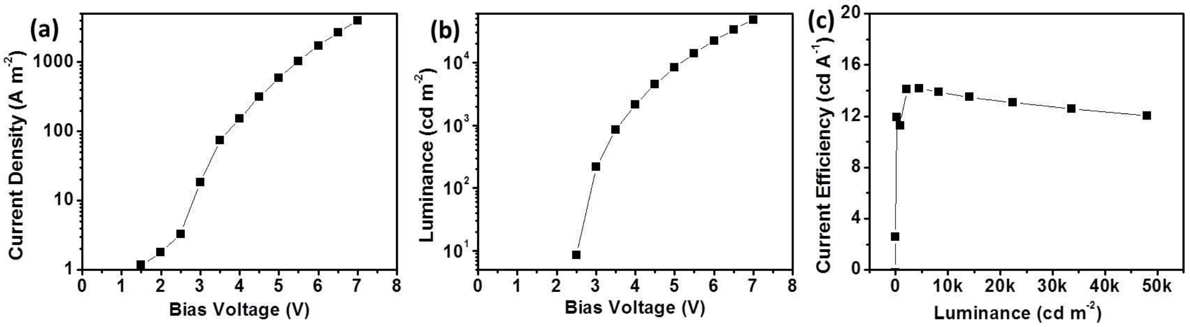

Supplementary Figure 12. Current density-bias voltage (a), luminance-bias voltage (b) and current efficiency-luminance (c) characteristics of white light PLEDs. Turn-on voltage is 2.5 V.

Supplementary Table 1. Resilience values for the EDC film with various strain cycles.

Strain cycles	10%	20%	30%
1	98.3%	94.7%	86.6%
2	99.9%	94.5%	91.6%
3	98.6%	95.4%	92.3%
4	99.1%	95.5%	92.2%
5	97.7%	95.8%	92.4%

Supplementary Note 1. Properties of the elastomeric dielectric copolymer

The siliconized urethane acrylate oligomer (CN990, obtained from Sartomer) was selected as main chain for its high stretchability and compatibility with the source/drain and gate composite electrode. The polyethylene glycol diacrylate (SR259) was selected as main for its high flexibility, low monomer viscosity (25 cps @ 25°C) which is important for printing, and high dielectric constant [1,2] and semi-crystalline structure of its homopolymer [1]. It has been reported that PEG with a molecular weight higher than 1,500 has a semi-crystalline structure [1]. The introduction of PEG with semi-crystalline structure into the polymer main chain is thus able to provide an internal surface between the amorphous and the crystalline phases, which is sufficient for Maxwell–Wagner–Sillars-type polarization [3]. The interfacial polarization of Maxwell–Wagner–Sillars-type polarization caused by trapping of free charges at the boundaries between the crystalline structure and the amorphous regions can result in the enhancement of dielectric constant for the PU-*co*-PEG copolymer. Moreover, the methoxy polyethylene glycol monomethacrylate (SR550) was selected as side chain for its high polarity and high chain mobility under room temperature. The methoxy polyethylene glycol monomethacrylate can act as a polar and flexible side chain, and thus can contribute a lot to improve the dielectric constant of the copolymer.

Various weight ratios of CN990:SR550:SR259 were studied, and a ratio of 1:1:1 was found to give the optimal overall performance of the resulting elastomeric dielectric copolymer (EDC) in terms of optical transmittance, dielectric constant, and mechanical compliance. The EDC has a dielectric constant about 13.0 and 8.75 at the frequency of 12 Hz and 1,000 Hz respectively, which is higher than that of the PU-*co*-PEG copolymer with a CN990:SR550:SR259 ratio of 1:0.5:1 and 1:0:1 (Supplementary Figure 2). A strong frequency-dependent behavior of the

dielectric constant for the PU-*co*-PEG copolymer was observed in the low frequency region (<50 Hz) followed by a nearly frequency-independent behavior at high frequency. The existence of the amorphous and semi-crystalline structure in the PU-*co*-PEG copolymer is the main reason for this frequency-dependency phenomenon [1]. The time required for the dipole polarization at the boundaries between the crystalline and amorphous regions is reduced as the frequency increases, and thus the dielectric constant decreases with the frequency increasing.

EDC exhibits an elongation at break of approximately 50% (Supplementary Figure 3(b)). Repeated loading and unloading strain cycles in standard tensile testing format were also applied on the EDC film to investigate its elasticity, hysteresis and resilience. Supplementary Figure 3(c) Figure shows the fifth loading and unloading cycles for EDC with strains value up to 30% (only the fifth cycle is presented for simplicity). The resilience value of the EDC film, as listed in Supplementary Table 1, was calculated by the area from the unloading curve by that of the loading curve. The almost overlap of the stress-strain curves during loading and unloading and the high resilience value (>90%) suggest the small hysteresis, fast recovery and good elasticity of the gate dielectric elastomer at strains up to 30% [4]. Moreover, the EDC film is provided with a very low loss factor of ~0.011 (Supplementary Figure 3(d)), another indication of small mechanical hysteresis, which is in favor of extending the cycling lifetime and improving the performance during cyclic deformation. This EDC film also exhibits a transmittance higher than 90% in the wavelength range from 400 to 1,000 nm (Supplementary Figure 3(a)). The morphology of the EDC layers on the AgNW-PUA composite source/drain electrodes was examined on an atomic force microscopy (AFM) as shown in Supplementary Figure 3(e). EDC has smooth surface, exhibiting an average roughness (*R*a) of 1.467 nm. Moreover, no pinholes or defects are observed on both dielectric layers.

Supplementary Part 2. Contact resistance between AgNW and SWCNT

The contact resistance between the semiconducting SWCNT network and AgNW electrodes has been characterized by transmission line model (TLM) measurements. An optical microscopy image of TLM test pattern of AgNWs is shown in Supplementary Figure 4(b). The test structure consists of a set of AgNW electrode contacts with identical geometry (width W = 1,000 μm and length D = 500 μm) and different spacing (50 μm, 80 μm, 100 μm, 120 μm, 140 μm, and 160 μm). Supplementary Figure 4(a) shows a plot of measured resistance between AgNW contacts at different spacing. According to the TLM theory [5,6], R is related to the SWCNT/AgNW contact resistance R_C and to the SWCNT sheet resistance R_S according to the equation: $R = 2R_C + R_S \cdot L/W = 2\, L_T \cdot R_S / W + R_S \cdot L/W$, where L_T is the transfer length defining average distance that an electron (or hole) travels in the semiconductor beneath the contact before it flows up into the contact. By linear fitting of the data in Supplementary Figure 4(a) by the Equation above, the contact resistance between SWCNT and AgNWs (R_C = 7,835 ± 1,565 Ω) contributions can be determined. The L_T can also be calculated to 5.6 μm. Since R_C clearly depends on the pad size, the specific contact resistance (contact resistivity) ρ_C, independent of contact area, is an critical parameter used to evaluate the quality of metal contact. According to the relation $R_C = \rho_C / L_T \cdot W = R_S \cdot L_T / W$, the contact resistivity ρ_C = 0.219 ± 0.043 $\Omega \cdot cm^2$ is calculated.

Supplementary Note 3. Hysteresis analysis of the SWCNT-AgNW TFTs

Hysteresis was observed in the I_D-V_G characteristics at various sweep rates and V_D as shown in Supplementary Figure 5. The width of the hysteresis in V_G exhibited a significant dependence on the sweeping rate of the gate voltage. The hysteresis increases as the sweep rate of V_G increases. Two reasons are suggested to cause the hysteresis in the SWCNT-AgNW TFT devices. 1) Water, commonly absorbed on the hydrophilic PEG in the EDC and the nanotube surface, can act as interfacial defects and are considered to be the main reason lead to hysteresis of the SWCNT-AgNW TFT [6-9]. Charging and discharging of the -OH groups in water by carriers in the channel region electrostatically modulate the SWNTs, thereby changing their conductivity. These –OH groups trap electrons under a positive bias on the gate and are discharged under a negative bias. 2) The relative large gate dielectric thickness (~1.1 μm) is also likely lead to the hysteresis in this SWCNT-AgNW TFT device.

Supplementary Note 4. Gate capacitance of SWCNT-AgNW TFTs

Gate capacitance is an essential parameter for extract the mobility of transistors. There are two models to calculate the gate capacitance of SWCNT network: (a) parallel plate model and (b) cylindrical model.

(a) Parallel plate model:

This model treats the nanotube film as a uniform film (ignore the edge effects) and determines the gate capacitance only depends on the thickness of the gate dielectric layer. We measured the capacitance of the DEC from parallel plate test structures using a LCR meter. The specific gate

capacitance of the DEC film was measured to 16.7 nF cm^{-2} at 12 Hz, and 11.0 nF cm^{-2} at 1,000 Hz.

(b) Cylindrical model:

However, SWCNTs are sparsely distributed in the channel, and the parallel-plate capacitor model overestimates the gate capacitance and underestimates the mobility. The other more sophisticated and rigorous model (cylindrical model) is to consider the electrostatic coupling between nanotubes, so that the capacitance per unit area can be calculated by applying the following equation [10]:

$$Ci = \left\{C_Q^{-1} + \frac{1}{2\pi\varepsilon_0\varepsilon_i}\ln\left[\frac{\Lambda}{R}\frac{\sinh(2\pi t_i/\Lambda)}{\pi}\right]\right\}^{-1}\Lambda^{-1} \qquad (1)$$

where C_Q is the quantum capacitance of carbon nanotubes (4.0×10^{-10} F m^{-1}) [11], Λ^{-1} stands for the linear density of nanotubes, $\varepsilon_o = 8.85\times10^{-12}$ F m^{-1} is the dielectric constant in vacuum, R = 1.5 nm (obtained from Nanointegis Inc.) is the radius of nanotubes, and t_i = 1.1 µm is the thickness of the dielectric layer. Based on the equation, one can find that C_i values were 10.5, 9.78 and 8.90 nF cm^{-2} as density of ~25, 13 and 6.0 tubes $µm^{-1}$. Thus, the cylindrical model yields an average mobility of 42.6, 27.1 and 17.3 cm^2 V^{-1} s^{-1} as density of ~25, 13 and 6.0 tubes $µm^{-1}$.

Supplementary Note 5. Device performance as a function of SWCNT density

Electrical characteristics of a typical SWCNT-AgNW TFT with a density of SWCNT about 13 tubes $µm^{-1}$ are shown in Supplementary Figure 6. An unstretched device mobility of 14.2 cm^2 V^{-1} s^{-1} is calculated from the transfer characteristics in Supplementary Figure 6(b). In 7 different

batches for a total of 33 TFT devices fabricated, the yield was 78.6%. The histograms of the statistical variations for all these devices in mobility, *g*m/*W*, I_{ON}/W, and $I_{ON/OFF}$ are displayed in Supplementary Figure 6(c) to 6(f). The mean of mobility, *g*m/*W*, I_{ON}/W, and $I_{ON/OFF}$ is calculated to be 15.9±6.34 $cm^2 V^{-1} s^{-1}$, 3.89±1.7 µS mm^{-1}, 12.9+5.5 µA mm^{-1} and 14,000±6,500.

Electrical characteristics of a typical SWCNT-AgNW TFT with a density of SWCNT about 6.0 tubes μm^{-1} are shown in Supplementary Figure 7. An unstretched device mobility of 8.05 $cm^2 V^{-1} s^{-1}$ is calculated from the transfer characteristics in Supplementary Figure 7(b). In 7 different batches for a total of 37 TFT devices fabricated, the yield was 88.1%. The histograms of the statistical variations for all these devices in mobility, *g*m/*W*, I_{ON}/W, and $I_{ON/OFF}$ are displayed in Supplementary Figure 7(c) to 7(f). The mean of mobility, *g*m/*W*, I_{ON}/W, and $I_{ON/OFF}$ is calculated to be 9.22±4.37 $cm^2 V^{-1} s^{-1}$, 1.51±0.78 µS mm^{-1}, 4.54+2.43 µA mm^{-1} and 18,300±8,100.

Supplementary Figure 8 shows I_{ON}/W, g_m/W, $I_{ON/OFF}$, and mobility as a function of density of nanotubes for V_D= -2.0 V. These device performances were dominated by the SWCNT density. It's clearly seen that by increasing the SWCNT density, the $I_{ON/OFF}$ decreased, whereas the I_{ON}/W, g_m/W and mobility were enhanced. As can be observed from Supplementary Figure 8(e), the increase in I_{ON} for the device with high SWCNT density is achieved at the expense of a high I_{OFF} which is mainly caused by inversion to n-type conduction. This relative high I_{OFF} was observed in all 36 devices with SWCNT density of 25 tubes μm^{-1}. The inversion to n-type conduction at low V_G accompanied by a high I_{OFF} is consistent with the behavior of a narrow band gap semiconductor and metallic nanotubes [12]. At high SWCNT densities, the density of metallic SWCNTs can exceed percolation threshold and the network will forms continuous paths across the device, which results in high I_{OFF} and low $I_{ON/OFF}$.

Supplementary Note 6. Stretchability of the EDC

Supplementary Figure 11(a) presents the strain-induced changes in capacitance of the EDC. It is interesting to note that the capacitance of EDC decreased as strain increased. This result contrasts with the previous studies in which the strain-induced capacitance of typical elastomer (e.g. polyurethane) increases with the strain increases [13]. We suggest this is mainly thanks to the special structure of the EDC. As discussed above, the PEG side chains and main chains should contribute a lot to the dielectric constant due to their high polarity and chain mobility under ambient conditions. However, stretching the EDC would 1) lead to stretch and align the molecular chains and thus increase interaction force among the molecular chains, and 2) decrease the chain mobility of the polar side chains. Both can result in the decrease of dielectric constant. Supplementary Figure 11(b) shows that the capacitance remained constant throughout the cycling measurements.

Supplementary Note 7. Device fabrication and performance of white light polymer OLED

White light polymer OLEDs based on ITO/glass anode and evaporated aluminum anode was fabricated according to previously reported work [14]. The ITO electrode was first pre-cleaned and treated with oxygen plasma to increase the work function and decrease the surface roughness. Poly(3,4-ethylenedioxythiophene):poly(styrenesulfonate) (PEDOT:PSS) (Clevios VP Al 4083 from H. C. Starck Inc.) was spin-coated on the ITO glass substrates at 3000 rpm for 60 s and then baked at 120 ^{o}C for 15 min. The resulting coating was approximately 40 nm thick. A white

emissive polymer (WP-2, Cambridge Display Technology) blend layer was then spin-coated from 1,3-bis[(4-tert-butylphenyl)-1,3,4-oxidiazolyl]phenylene (OXD-7, Lumtech), co-dissolved in chlorobenzene. The emissive layer, 75 nm in thickness, was annealed at 80 ^{o}C for 30 min. Finally, a thin layer of CsF (1.0 nm) and a layer of aluminum (100 nm) were successively deposited in a vacuum thermal evaporator through a shadow mask at 10^{-6} Torr.

Supplementary Figure 12 shows the current density-bias voltage, luminance-bias voltage, and current efficiency versus luminance characteristics of the typical while light PLED fabricated on ITO/glass anode and Al cathode. The turn-on voltage of the device is ~ 2.5 V, and the maximum current efficiency of the device is 14.2 cd A^{-1} at the brightness of 4,400 cd m^{-2}.

Supplementary References:

1. Liu, H. L. *et al.* A new kind of electro-active polymer composite composed of silicone elastomer and polyethylene glycol. *J. Phys. D: Appl. Phys.* **45**, 485303 (2012).

2. Mali, C. S., Chavan, S. D., Kanse, K. S., Kumbharkhane, A. C. and Mehrotra, S. C. Dielectric relaxation of poly ethylene glycol–water mixtures using time domain technique. *Indian J. Pure Appl. Phys.* **45**, 476–481 (2007).

3. Alig, I., Dudkin, S. M., Jenninger, W. and Marzantowicz, M. 2006 Ac conductivity and dielectric permittivity of poly(ethylene glycol) during crystallization: percolation picture *Polymer* **47**, 1722–1731(2006).

4. Li, L. Q., Teller, S., Clifton, R. J., Jia, X. Q. & Kiick, K. L. Tunable Mechanical Stability and Deformation Response of a Resilin-Based Elastomer. *Biomacromolecules*, **12**, 2302–2310 (2011).

5. Xu, H., Chen, L., Hu, L. B. & Zhitenev, N. Contact resistance of flexible, transparent carbon nanotube films with metals. *Appl. Phys. Lett.* **97**, 143116 (2010).

6. Jackson, R. & Graham, S. Specific contact resistance at metal/carbon nanotube interfaces. *Appl. Phys. Lett*. **94**, 012109 (2009).

7. Jin, S. H. *et al.* Sources of Hysteresis in Carbon Nanotube Field-Effect Transistors and Their Elimination Via Methylsiloxane Encapsulants and Optimized Growth Procedures. *Adv. Funct. Mater*. **22**, 2276–2284 (2012).

8. Kim , W. *et al.* Hysteresis Caused by Water Molecules in Carbon Nanotube Field-Effect Transistors. *Nano Lett*. **3**, 193-198 (2003).

9. Lee , J. S. *et al*. Origin of Gate Hysteresis in Carbon Nanotube Field-Effect Transistors. *J . Phys. Chem. C*, **111** , 12504-12507 (2007).

10. Cao, Q. et al. Gate capacitance coupling of singled-walled carbon nanotube thin-film transistors. *Appl. Phys. Lett*. **90**, 023516 (2007).

11. Rosenblatt, S. et al. High performance electrolyte gated carbon nanotube transistor. *Nano Lett*. **2**, 869-872 (2002).

12. Snow, E. S., Novak, J. P., Campbell, P. M. and Park D. Random networks of carbon nanotubes as an electronic material. *Appl. Phys. Lett.*, **82**, 2145-2147 (2003).

13. Chortos, A. *et al.* Highly stretchable transistors using a microcracked organic semiconductor. *Adv. Mater.* **26,** 4253–4259 (2014).

14. Li, L. *et al.* A solution processed flexible nanocomposite electrode with efficient light extraction for organic light emitting diodes. *Sci. Rep.* **4,** 4307 (2014).
